# Androgen receptor activity in prostate cancer dictates efficacy of bipolar androgen therapy through MYC

**DOI:** 10.1172/JCI162396

**Published:** 2022-12-01

**Authors:** Laura A. Sena, Rajendra Kumar, David E. Sanin, Elizabeth A. Thompson, D. Marc Rosen, Susan L. Dalrymple, Lizamma Antony, Yuhan Yang, Carolina Gomes-Alexandre, Jessica L. Hicks, Tracy Jones, Kiara A. Bowers, Jillian N. Eskra, Jennifer Meyers, Anuj Gupta, Alyza Skaist, Srinivasan Yegnasubramanian, Jun Luo, W. Nathaniel Brennen, Sushant K. Kachhap, Emmanuel S. Antonarakis, Angelo M. De Marzo, John T. Isaacs, Mark C. Markowski, Samuel R. Denmeade

**Affiliations:** The Sidney Kimmel Comprehensive Cancer Center, Johns Hopkins University, Baltimore, Maryland, USA.

**Keywords:** Oncology, Prostate cancer

## Abstract

Testosterone is the canonical growth factor of prostate cancer but can paradoxically suppress its growth when present at supraphysiological levels. We have previously demonstrated that the cyclical administration of supraphysiological androgen (SPA), termed bipolar androgen therapy (BAT), can result in tumor regression and clinical benefit for patients with castration-resistant prostate cancer. However, predictors and mechanisms of response and resistance have been ill defined. Here, we show that growth inhibition of prostate cancer models by SPA required high androgen receptor (AR) activity and were driven in part by downregulation of MYC. Using matched sequential patient biopsies, we show that high pretreatment AR activity predicted downregulation of MYC, improved clinical response, and prolonged progression-free and overall survival for patients on BAT. BAT induced strong downregulation of AR in all patients, which is shown to be a primary mechanism of acquired resistance to SPA. Acquired resistance was overcome by alternating SPA with the AR inhibitor enzalutamide, which induced adaptive upregulation of AR and resensitized prostate cancer to SPA. This work identifies high AR activity as a predictive biomarker of response to BAT and supports a treatment paradigm for prostate cancer involving alternating between AR inhibition and activation.

## Graphical abstract

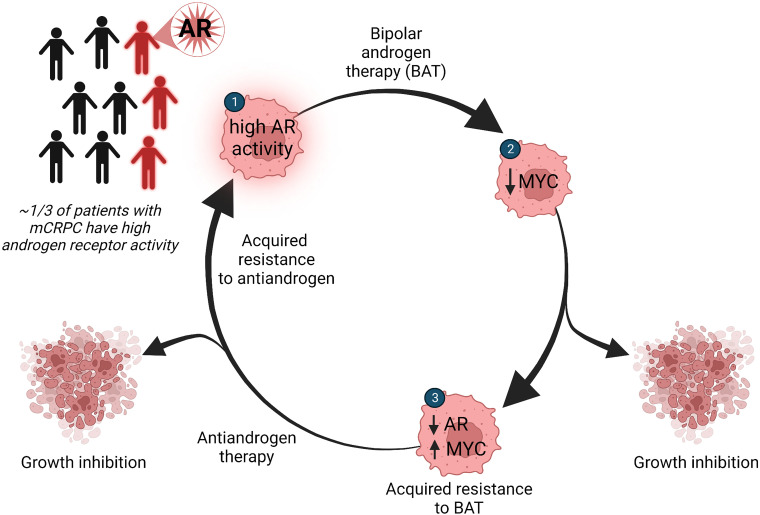

## Introduction

Signaling through the androgen receptor (AR) is the primary oncogenic driver of prostate adenocarcinoma. Inhibition of AR signaling by androgen deprivation and AR inhibitors produces significant therapeutic and palliative benefit and constitutes the cornerstone of treatment for advanced disease. Yet this therapeutic strategy is not curative, and patients eventually progress with lethal castration-resistant prostate cancer (CRPC). Studies dating back over 30 years indicate that supraphysiological androgens (SPAs) can paradoxically suppress the growth of some prostate cancer cell lines and xenograft models (1–3). These studies led us to test SPA as a treatment for men with CRPC. Our approach has been to pulse intramuscular testosterone every 28 days concurrent with ongoing administration of luteinizing hormone-releasing hormone analogue to result in the oscillation of serum testosterone from supraphysiological to near-castrate level. Given this oscillation of testosterone between polar extremes, we termed this treatment bipolar androgen therapy (BAT) (4). We have previously described that BAT can produce clinical benefit and tumor regression for 20%–30% of patients with CRPC (5–9); however, predictors and mechanisms of response and resistance have been ill-defined. A better understanding of the molecular mechanisms of BAT is needed both to optimize patient selection and to develop strategies to enhance its therapeutic efficacy.

Early studies of SPA-mediated growth inhibition of prostate cancer models suggested that this effect occurs predominantly after long-term exposure to low androgen conditions and requires treatment with supraphysiological, rather than physiological, doses of androgens (2). Thus, it seems that an adaptation of prostate cancer to inhibition of AR signaling constitutes a vulnerability to SPA. Major adaptations of prostate cancer to the inhibition of AR signaling are counterbalanced enhancement of AR signaling through AR overexpression, gene amplification, activating mutations, and expression of ligand-independent variants (10, 11). Indeed, an association between high AR expression and growth-inhibition by SPA in prostate cancer models has been observed (2, 12, 13), begging the question of whether high AR activity is necessary for prostate cancer sensitivity to SPA.

We and others are working to map the downstream molecular events through which SPA results in growth inhibition of SPA-sensitive prostate cancer models. Multiple mechanisms have been proposed, including downregulation of c-MYC (hereafter called MYC) (1, 13–15), AR inhibition of DNA relicensing during mitosis (16), AR-mediated DNA damage (12, 17), and induction of ferroptosis and immunogenic cell death (18). Notably, in vitro genome-wide positive-selection genetic screens have failed to identify 1 dominant pathway mediating growth inhibition by SPA (19), suggesting that SPA likely induces multiple parallel growth-inhibitory pathways in SPA-sensitive prostate cancer. However, the clinical significance of the multiple proposed mechanisms of growth inhibition by SPA has been unclear, as no study has utilized tumor samples derived from patients treated with SPA to date. Given that c-MYC (here-after called MYC) is highly expressed in prostate cancer (20, 21), is a potent driver of growth and proliferation (22), and yet has been described as ‘undruggable’ (23), we have been particularly interested in determining whether BAT can reduce MYC expression and understanding the relative contribution of downregulation of MYC toward growth inhibition by SPA.

Here, we utilize both paired sequential biopsies of metastases of patients with CRPC who have been treated with BAT and prostate cancer models to identify predictors and molecular mechanisms of prostate cancer regression by BAT. We report that high AR activity is required for growth inhibition of prostate cancer models by SPA, which occurs, in part, through downregulation of MYC. Similarly, in patients, high pretreatment AR activity predicts the downregulation of MYC, tumor regression, and both progression-free and overall survival on BAT. Notably, we found that acquired resistance to SPA could be driven by the downregulation of AR but that this could be overcome by treatment with an AR inhibitor, which induced adaptive upregulation of AR.

## Results

### AR activity determines response to SPA in vitro.

**Figure 1:**
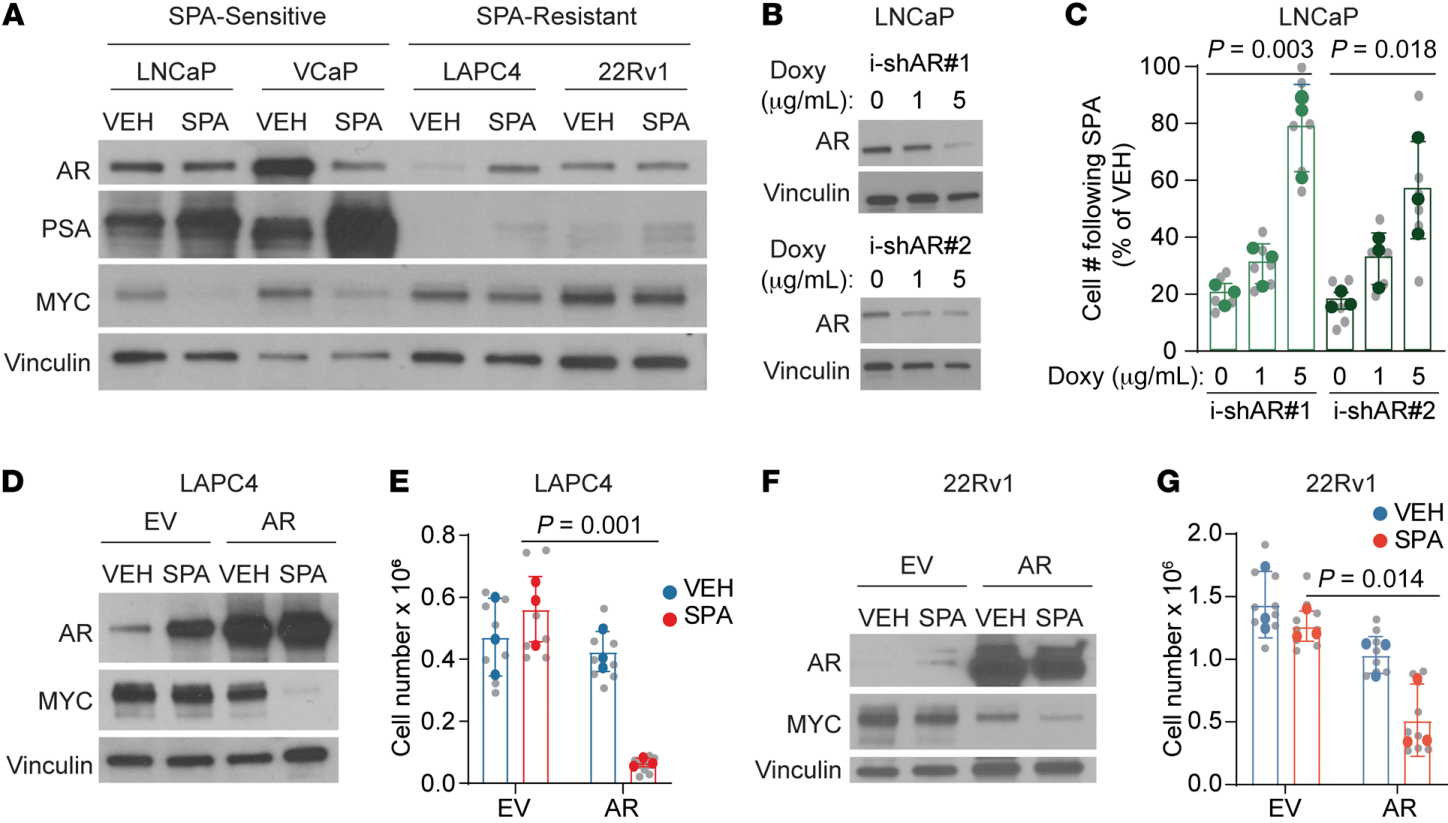
High pretreatment AR activity is required and sufficient for growth inhibition by SPA. (**A**) AR, PSA, and MYC protein expression by Western blot of prostate cancer cell lines treated with VEH or SPA for 72 hours. Representative blot of *n* = 3 independent experiments. (**B**) AR protein expression by Western blot of LNCaP cells expressing doxycycline-inducible shRNA against AR pretreated with the indicated concentration of doxycycline (doxy) for 72 hours. Representative blot of *n* = 2 experiments. (**C**) Viable cell counts of LNCaP-shAR pretreated with indicated concentration of doxycycline for 72 hours then VEH or SPA for 96 hours (*n* = 3 independent experiments). (**D**) AR and MYC protein expression by Western blot of LAPC4 expressing empty vector (EV) or AR treated with VEH or SPA for 7 days. Representative blot of *n* = 3 independent experiments. (**E**) Viable cell counts of LAPC4-EV and LAPC4-AR cell lines treated with VEH or SPA for 7 days (*n* = 3 independent experiments). (**F**) AR and MYC protein expression by Western blot of 22Rv1 cells expressing empty vector (EV) or AR treated with VEH or SPA for 4 days. Representative blot of *n* = 3 independent experiments. (**G**) Viable cell counts of 22Rv1-EV and 22Rv1-AR cell lines treated with VEH or SPA for 7 days (*n* = 3 independent experiments). VEH, vehicle control EtOH 0.01%. SPA, R1881 10nM. (**C**, **E**, **G**) *P* value by unpaired 2-tailed *t* test comparing final cell counts. Biological replicates indicated in gray with mean of each independent experiment in color. i-shAR, inducible-short hairpin RNA against AR. For Western blots, vinculin was used as a loading control.

To assess determinants of sensitivity to SPA, we first studied human prostate cancer cell lines with varying sensitivity to SPA. SPA can be provided to cells as R1881, a potent synthetic androgen that is not metabolized in vitro, at a dose of 10 nM, which is approximately 20-fold higher than the level of free testosterone in eugonadal adult men. LNCaP and VCaP cell lines are growth inhibited by SPA, while LAPC4 and 22Rv1 cell lines exhibit primary resistance to SPA (Supplemental Figure 1; supplemental material available online with this article; https://doi.org/10.1172/JCI162396DS1). Among these cell lines, we observed that pretreatment AR abundance and activity (as assessed by expression of the AR target prostate-specific antigen (PSA)) was higher in SPA-sensitive cell lines than SPA-resistant cell lines (Figure 1A). High baseline AR abundance and activity was required for growth inhibition by SPA, as inducible shRNA-mediated knock-down of AR in LNCaP cells (Figure 1B) resulted in resistance to SPA (Figure 1C) and rescued clonogenic survival following SPA treatment (Supplemental Figure 2). Conversely, high AR abundance was sufficient to confer sensitivity to SPA, as overexpression of AR in LAPC4 and 22Rv1 cells (Figure 1, D and F) resulted in growth inhibition by SPA (Figure 1, E and G). This indicates that AR abundance and activity is a major determinant of prostate cancer response to SPA in vitro.

### Downregulation of MYC contributes to growth inhibition by SPA.

**Figure 2:**
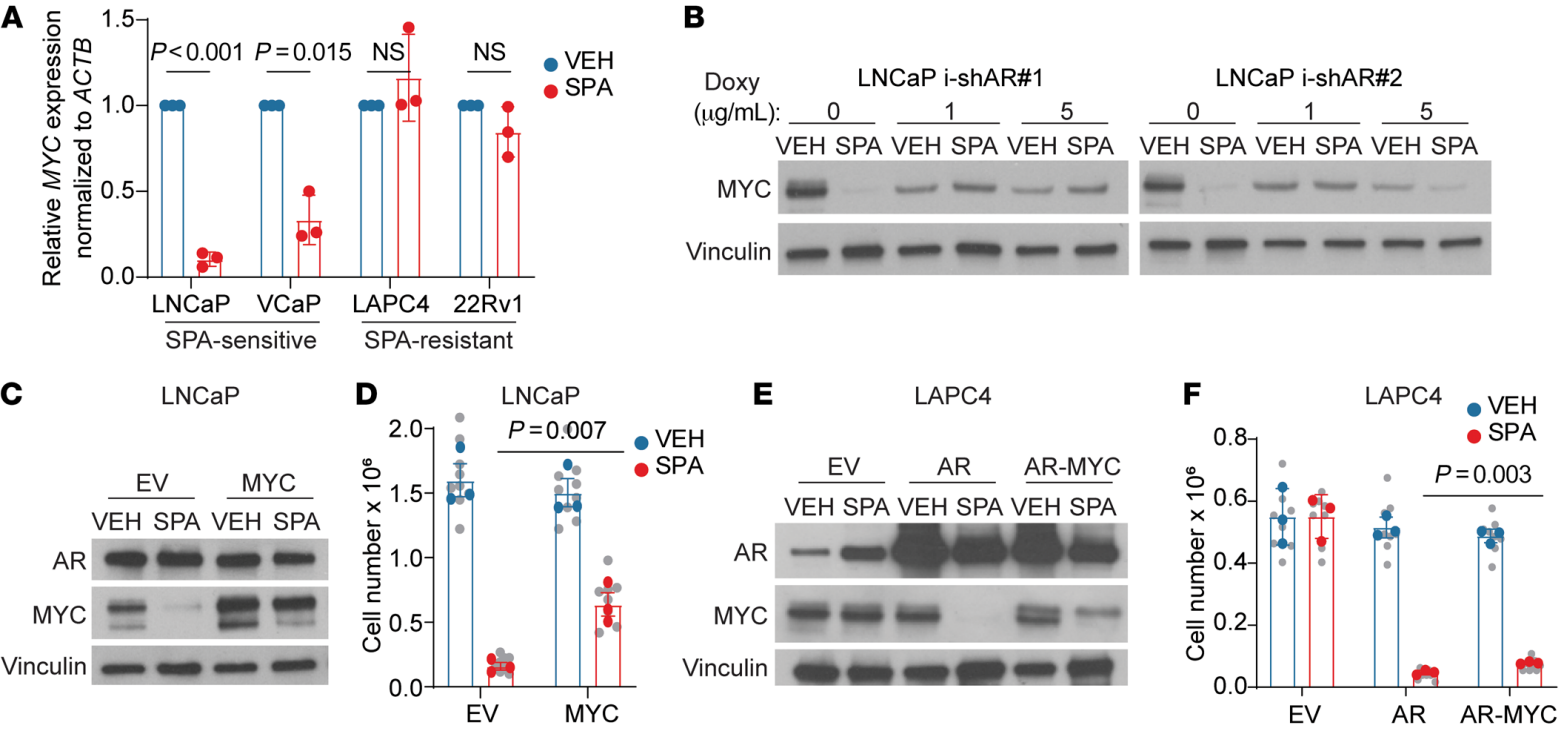
High pretreatment AR activity is required for downregulation of MYC by SPA, which contributes to growth inhibition. (**A**) *MYC* mRNA expression by quantitative PCR (qPCR) of prostate cancer cell lines treated with VEH or SPA for 72 hours (*n* = 3 independent experiments). Ct value was first normalized to *ACTB* for each sample, then to VEH for each cell line, and expressed as mean ± SD with *P* values were determined by unpaired 2-tailed *t* test with Welch correction for unequal variances. (**B**) MYC protein expression by Western blot of LNCaP cells expressing doxycycline-inducible shRNA against AR pretreated with indicated concentration of doxycycline (doxy) for 72 hours then VEH or SPA for 96 hours. Representative blot of *n* = 2 experiments. (**C**) AR and MYC protein expression by Western blot of LNCaP-empty vector (LNCaP-EV) and LNCaP-MYC cell lines treated with VEH or SPA for 72 hours. Representative blot of *n* = 3 independent experiments. (**D**) Viable cell counts of LNCaP-EV and LNCaP-MYC cell lines treated with VEH or SPA for 7 days (*n* = 3 independent experiments). *P* value was determined by unpaired 2-tailed *t* test. Biological replicates are indicated in gray with the mean of each independent experiment in color. (**E**) AR and MYC expression by Western blot of LAPC4-EV, LAPC4-AR, and LAPC4-AR-MYC cell lines treated with VEH or SPA for 7 days. Representative blot of *n* = 3 independent experiments. (**F**) Viable cell counts of LAPC4-EV, LAPC4-AR, and LAPC4-AR-MYC cell lines treated with VEH or SPA for 7 days (*n* = 3 independent experiments). *P* value was determined by unpaired 2-tailed *t* test. Biological replicates indicated in gray with the mean of each independent experiment in color. VEH, vehicle control, EtOH 0.01%. SPA, R1881, 10nM. For Western blots, vinculin was used as a loading control.

AR activation has previously been shown to downregulate MYC in normal prostate epithelial cells (24–26) and models of prostate cancer (1, 13–15). We observed that SPA downregulates MYC, but only in cells lines with high AR abundance and activity (i.e., SPA-sensitive cell lines) and not in SPA-resistant cell lines (Figure 1A and Figure 2A). High pretreatment AR abundance was required for downregulation of MYC by SPA, as inducible shRNA-mediated knock-down of AR in LNCaP cells disabled MYC downregulation by SPA (Figure 2B). Moreover, high pretreatment AR abundance was sufficient to induce downregulation of MYC by SPA, given that AR overexpression resulted in MYC downregulation in LAPC4 and 22Rv1 cells (Figure 1, D and F). As previously shown (2, 13), constitutive expression of MYC partially rescued growth inhibition in LNCaP and LAPC4-AR cells treated with SPA (Figure 2, C–F), indicating that MYC downregulation contributed to growth inhibition induced by SPA. Notably, downregulation of MYC is not a general feature of prostate cancer growth arrest, as the highly active chemotherapy agent docetaxel did induce growth arrest of LNCaP cells but did not cause downregulation of MYC (Supplemental Figure 3, A and B). Thus, downregulation of MYC is a specific feature of growth inhibition by SPA. Altogether, these data suggest that SPA inhibits growth of prostate cancer with high AR abundance in part through downregulation of MYC.

### AR activity determines response to BAT.

**Figure 3:**
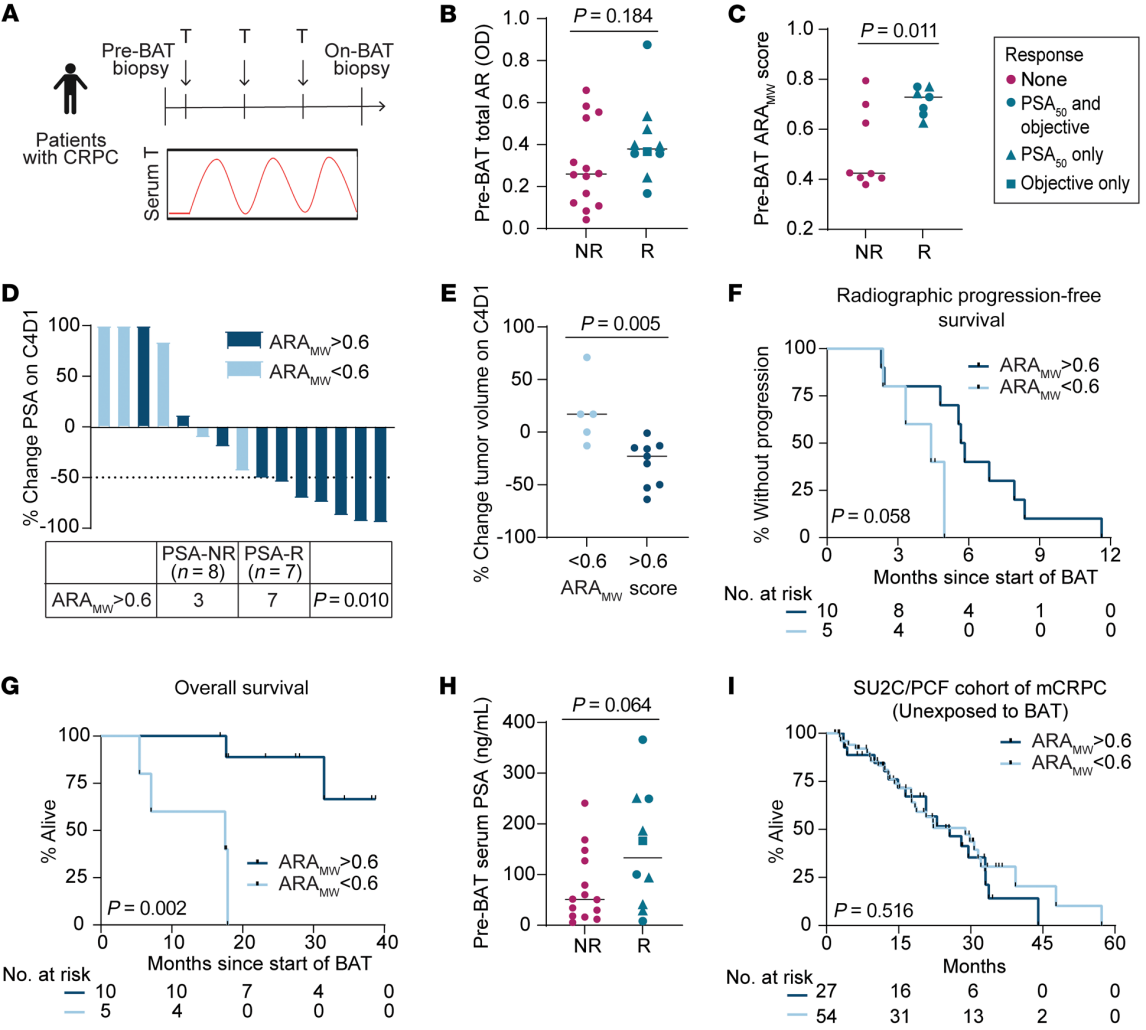
High pretreatment AR activity predicts clinical benefit from BAT. (**A**) Clinical trial design. CRPC, castration-resistant prostate cancer. BAT, Bipolar Androgen Therapy. T, testosterone. (**B**) PreBAT total AR OD by image analysis among nonresponders (NR) and responders (R) with median indicated by line (*n* = 24). Responders are those with a PSA_50_ response or objective response on C4D1. *P* values determined by unpaired 2-tailed *t* test. (**C**) PreBAT ARA_MW_ score among NR and R with median indicated by line (*n* = 15). *P* values determined by unpaired 2-tailed *t* test. (**D**) Percent change in PSA on C4D1 color-coded by ARA_MW_ score. PSA_50_ response indicated by dashed line. p value by Chi-squared comparison of proportions. (**E**) Percent change in tumor volume on C4D1 by ARA_MW_ score. *P* values determined by unpaired 2-tailed *t* test. (**F**) Radiographic progression-free survival on BAT stratified by ARA_MW_ score. *P* value determined by log-rank. (**G**) Overall survival on BAT stratified by ARA_MW_ score. *P* value determined by log-rank. (**H**) PreBAT serum PSA among NR and R with median indicated by line (*n* = 24). *P* value determined by unpaired 2-tailed *t* test. (**I**) Overall survival of patients in the SU2C/PCF cohort (*n* = 81) stratified by ARA_MW_ score. *P* value determined by log-rank.

To assess molecular mechanisms of BAT in patients with metastatic CRPC (mCRPC), we evaluated clinical samples from a prospective clinical trial (NCT03554317) that included on-study sequential paired biopsies of soft tissue metastases before (preBAT) and after 3 cycles of BAT (on-BAT) (Figure 3A). Twenty-four patients had tumor samples collected at both time points that were adequate for IHC analysis, and 15 of these patients had paired samples adequate for RNA sequencing analysis Of the 24 patients, 10 were considered to be responders, based on the presence of a decline in the serum PSA by at least 50%, or a decrease in tumor volume by at least 30% on day 1 of cycle 4 of BAT. Characteristics of these patients are listed in Supplemental Table 1. We first quantified AR protein abundance via IHC after performing serial dilutions of the AR antibody to ensure staining in the linear range. To separately measure AR in the nucleus, cytoplasm, and whole cell, we developed an iterative multiplex assay using AR and keratin 8. These were used to help train a random forest classifier to segment total tumor cellular area. In this manner, by image analysis, we were able to obtain OD measurements as a continuous variable separately for the nuclei, cytoplasm, and whole cell. In preBAT samples, responders did not exhibit higher total cellular AR protein abundance than nonresponders (Figure 3B). This directly correlated with AR protein abundance in the cytoplasm and the nucleus, as well as *AR* mRNA abundance (Supplemental Figure 4, A–C). Prior to BAT, the nuclear-to-cytoplasmic ratio of AR was greater than 1 in all patients, indicating that the majority of AR resided in the nucleus in advanced mCRPC, which was not significantly different between responders and nonresponders (Supplemental Figure 4D). To examine whether there was greater variation in pretreatment AR activity between responders and nonresponders, we generated an AR activity score using Mann-Whitney ranking of expression of 10 canonical AR target genes (ARA_MW_ score) (see Supplemental Methods). Notably, responders had significantly higher preBAT ARA_MW_ scores than nonresponders (*P* = 0.011) (Figure 3C). The ARA_MW_ score was not driven by expression of 1 dominant gene (Supplemental Figure 5A); the included gene transcripts did not exhibit significant colinearity (Supplemental Figure 5B); and the score did not correlate with AR protein abundance (Supplemental Figure 5C). Thus, each included gene contributed unique data to the ARAMW score, which was distinct from AR abundance. This demonstrates that AR activity in advanced CRPC is controlled by factors beyond protein abundance, which likely includes activating or inactivating gene mutations in AR and activity and abundance of AR cofactors and regulators (27, 28). Stratifying patients by a cutoff ARA_MW_ score of 0.6 (selected due to its ability to stratify patients with distinct outcomes), we observed that patients with high (>0.6) ARA_MW_ scores exhibited greater PSA responses (*P* = 0.010) (Figure 3D), greater decrease in tumor volume (*P* = 0.005) (Figure 3E), a trend toward longer radiographic progression-free survival (*P* = 0.058) (Figure 3F), and longer overall survival (*P* = 0.002) (Figure 3G) on BAT. Given that serum PSA concentration is, in part, dependent on cancer cell AR activity, we assessed whether there was an association between preBAT serum PSA and response to BAT. There was a trend toward higher preBAT serum PSA among responders compared with nonresponders (*P* = 0.064) (Figure 3H). Together, these data indicate that pretreatment AR activity is a major determinant of CRPC response to BAT, and the ARA_MW_ score may constitute a valuable predictive biomarker for this therapy.

Some strengths of the ARA_MW_ score are that it does not require a reference expression vector and is independent of variations in sequencing depth and processing. Therefore, we applied ARA_MW_ scoring to an independent cohort of 266 patients with mCRPC who were not exposed to BAT (SU2C/PCF cohort) (29). Among these patients, the prevalence of the biomarker (score >0.6) was 36.5% (Supplemental Figure 6A). Analysis of the SU2C/PCF cohort indicated that high AR activity is not independently prognostic — i.e., ARA_MW_ score >0.6 does not predict favorable outcomes independent of BAT treatment (Figure 3I) — and is not clearly associated with particular genomic alterations or other patient factors (Supplemental Figure 6, B–D).

### BAT downregulates MYC in responding patients.

**Figure 4:**
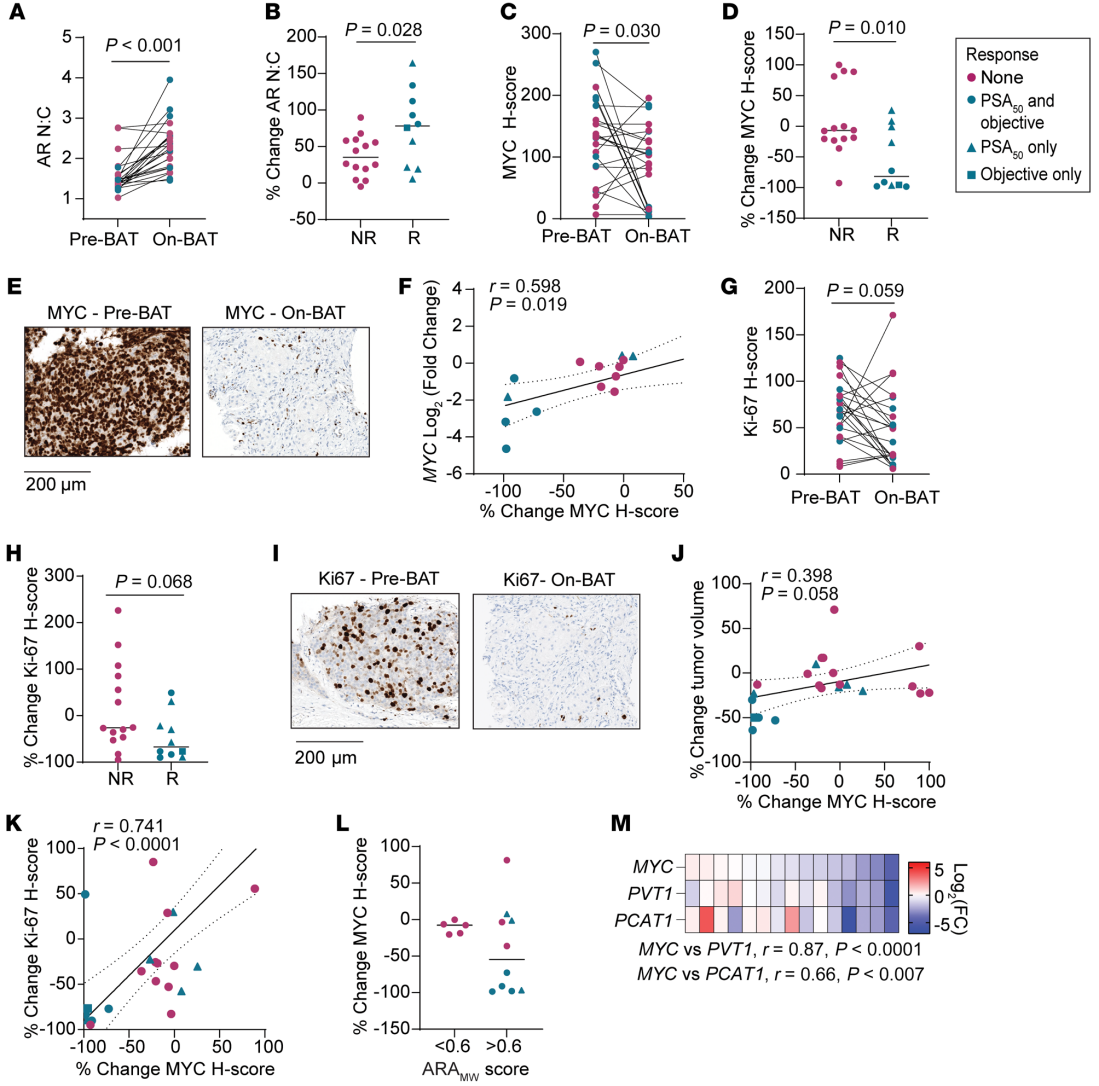
BAT downregulates MYC in responding patients. (**A**) AR nuclear-to-cytoplasmic ratio (AR N:C) in paired tumor biopsies (*n* = 24). (**B**) Percent change in AR N:C among nonresponders (NR) and responders (R) with median indicated by line. (**C**) MYC H-score in paired tumor biopsies (*n* = 24). (**D**) Percent change in MYC H-score among NR and R with median indicated by line. (**E**) Example of IHC for MYC in paired biopsy samples from a responding patient. Scale bar: 200 μm. (**F**) Correlation of *MYC* RNA change from C1D1 to C4D1 with MYC protein change from C1D1 to C4D1 (*n* = 15). (**G**) Ki-67 H-score in paired tumor biopsies (*n* = 24). (**H**) Percent change in Ki-67 H-score among NR and R with median indicated by line. (**I**) Example of IHC for Ki-67 in paired biopsy samples from a responding patient. (**J**) Correlation of percent change in tumor volume from C1D1 to C4D1 with MYC protein change from C1D1 to C4D1 (*n* = 23; 1 patient excluded for lack of measurable disease). (**K**) Correlation of percent change in Ki-67 H-score with percent change in MYC H-score (*n* = 24). (**L**) Percent change in MYC H-score stratified by ARA_MW_ score (*n* = 15) with median indicated by line. (**M**) Correlation of change in expression of genes within the 8q24 TAD with change in MYC expression (*n* = 15). (**A**, **C**, **G**) *P* value determined by paired 2-tailed *t* test. (**B**, **D**, **H**) *P* value determined by unpaired 2-tailed *t* test. (**F**, **J**, **K**, and **M**) *r* and *P* values determined by Pearson’s correlation calculation.

We next assessed molecular changes induced by BAT in the paired sequential tumor biopsies. BAT increased the AR nuclear-to-cytoplasmic ratio in most patients (Figure 4A), but to a notably greater degree in responders (Figure 4B). This may suggest that nuclear recruitment/retention and/or cytoplasmic clearance of AR is related to a clinical response to BAT. We also examined MYC expression by quantitative image analysis of MYC IHC. Most patients had high expression of MYC protein in the preBAT tumor sample (Figure 4C). BAT decreased the median MYC Histoscore (H-score) (Figure 4C), with responders exhibiting a greater decrease in the MYC H-score than nonresponders (Figure 4D) and a subset of patients having a near-complete ablation of MYC expression (Figure 4E). The change in MYC protein expression directly correlated with change in *MYC* mRNA expression (Figure 4F), suggesting that BAT suppressed MYC at the level of transcription and/or mRNA stability. BAT also decreased the median Ki-67 H-score (Figure 4G), with responders exhibiting a trend toward greater decrease in the Ki-67 H-score than nonresponders (Figure 4H), and some patients showing almost complete loss of Ki-67 (Figure 4I). The change in MYC protein expression directly correlated with the change in Ki-67 expression (Figure 4K) and the change in tumor volume on CT scan (Figure 4J). Notably, only patients with preBAT ARA_MW_ scores greater than 0.6 exhibited a significant decrease in MYC protein expression (Figure 4L), supporting the concept that high preBAT AR activity is required for downregulation of MYC and tumor regression by SPA.

The mechanism by which AR activation suppresses MYC in prostate cancer was recently suggested to occur through AR-mediated sequestration of cofactors and decreased activity of distal super enhancers (SE) near *PCAT1* that regulate the *MYC* promoter, as well as those of neighboring transcripts embedded in the topologically associated domain (TAD) on 8q24 (13). We noted that transcripts of the genes located within the 8q24 TAD, *PCAT1* and *PVT1*, had similar change in expression as *MYC* on BAT (r=0.87, *P <* 0.0001, and r=0.66, *P* = 0.007, respectively) (Figure 4M), which supports an idea that BAT reduces *MYC* mRNA expression via disruption of distal SE activity.

By principal component analysis of the RNA sequencing data, the differences in gene expression profiles between patients was generally much greater than the differences in gene expression induced by BAT (Supplemental Figure 7A). This interpatient heterogeneity of gene expression and the relatively small number of patients studied limited some analyses of the data. Notably, only 5 genes were identified to be statistically significantly altered by BAT, including downregulation of *AR*, *ANKRD30A*, and *LINC00993*, and upregulation of *RERGL* and *PARM1* (Supplemental Figure 7B). In contrast, numerous genes were differentially expressed between responders and nonresponders (Supplemental Figure 7C), however all of these genes were expressed at low levels (log_2_TPM less than 3), so the importance of these differences is uncertain.

Previous studies have suggested that SPA can induce DNA damage and downregulation of homologous recombination repair (HRR) gene expression in prostate cancer cell lines (12) and that patients with HRR genomic alterations may have heightened clinical responses to BAT (12, 30). In this data set, BAT did not significantly alter expression of a panel of HRR genes, nor was the change in expression of these genes different between responders and nonresponders (Supplemental Figure 8A). Additionally, there was no difference in clinical outcome based on the presence of a genomic HRR alteration identified through clinical testing among these patients (Supplemental Figures 8, C–F).

### Downregulation of AR drives acquired resistance to SPA.

**Figure 5:**
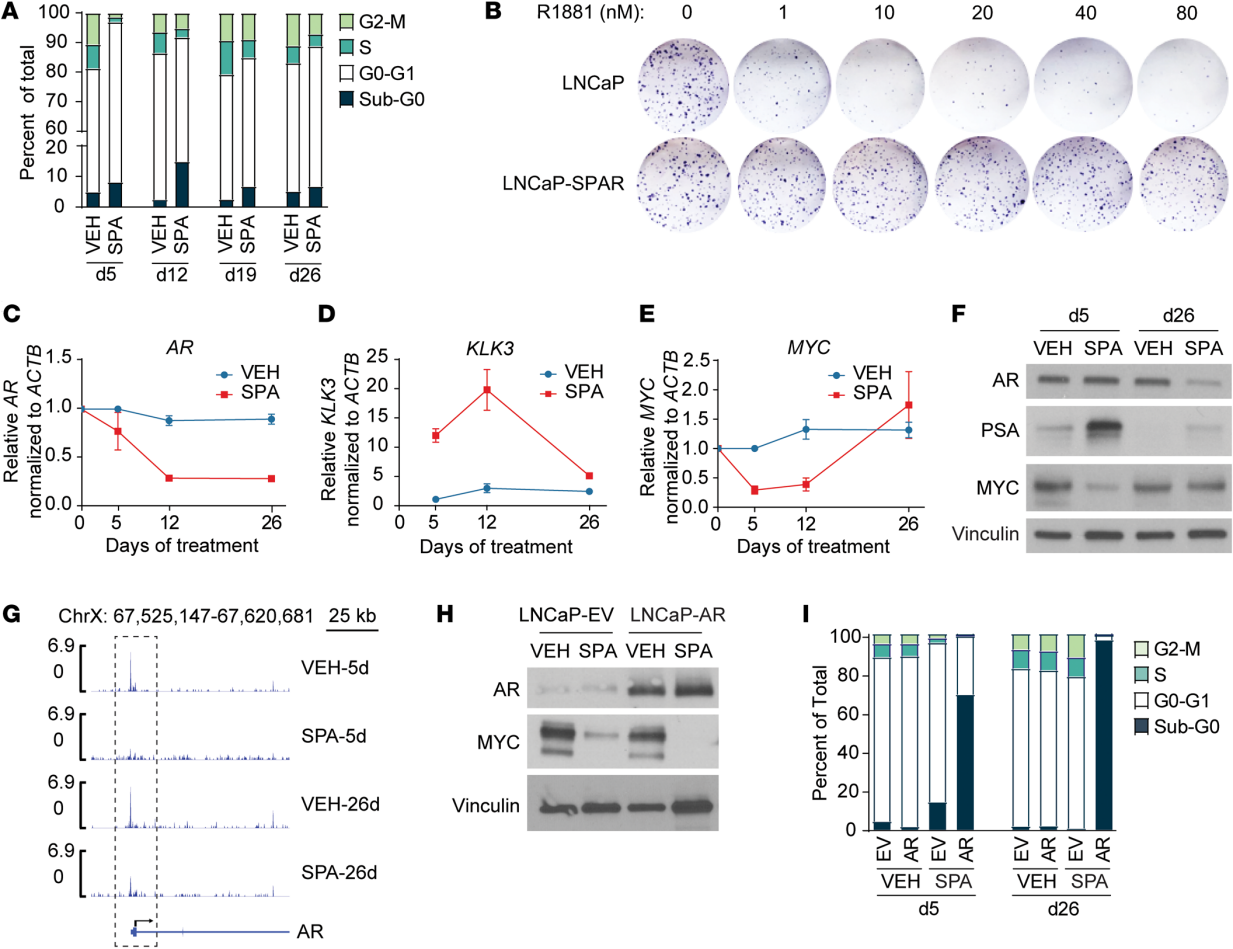
Downregulation of AR drives acquired resistance to SPA. (**A**) Cell cycle analysis by propidium iodide staining of LNCaP cells treated with VEH or SPA. Average values of *n* = 2 independent experiments. (**B**) Clonogenic survival of LNCaP cells treated for 26 days with VEH or SPA, followed by 7 days rest without treatment, then treatment with dose of R1881 as indicated. Representative photograph of *n* = 2 independent experiments. (**C**–**E**) *AR, KLK3,* and *MYC* mRNA expression by qPCR of LNCaP cells treated with VEH or SPA (*n* = 3 independent experiments). Ct values were normalized to *ACTB* for each sample, then to VEH × 5 days, and expressed as median ± SD with *P* values determined by unpaired 2-tailed *t* test. (**F**) AR, PSA, and MYC protein expression by Western blot of LNCaP cells treated with VEH or SPA for 5 or 26 days. Representative blot of *n* = 3 independent experiments. (**G**) Chromatin accessibility by ATAC-Seq of the AR promoter (dotted box) of LNCaP cells treated with VEH or SPA for 5 or 26 days (performed in duplicate). (**H**) AR and MYC protein expression by Western blot of LNCAP-EV and LNCAP-AR cells treated with VEH or SPA for 72 hours. Representative blot of *n* = 3 independent experiments. (**I**) Cell cycle analysis by propidium iodide staining of LNCaP-EV and LNCaP-AR cells treated with VEH or SPA. Average values of *n* = 3 independent experiments. VEH, vehicle control, EtOH 0.01%; SPA, R1881, 10nM; LNCaP-SPAR, LNCaP with acquired resistance to SPA. For Western blots, vinculin was used as a loading control.

Clinically, we have observed that most patients with CRPC who initially respond to BAT acquire secondary resistance after approximately 6–12 months of therapy (9). Similarly, the SPA-sensitive cell line LNCaP, which was initially cell cycle-arrested in G0–G1 after 5 days of SPA, reentered the cell cycle following 12–19 days of continuous SPA exposure (Figure 5A). Acquired resistance to SPA was verified in these cells, as retreatment with increasing doses of R1881 resulted in no change to clonogenic survival (Figure 5B). The transcriptional and chromatin accessibility profiles of LNCaP with acquired resistance to SPA (LNCaP-SPAR) appeared most similar to VEH-treated cells (Supplemental Figure 9, A and B), suggesting these cells revert to a pretreatment phenotype. Resistance did not appear to be driven by complete failure of SPA to activate AR, as hallmark androgen response genes remained induced in both SPA-sensitive and resistant cells (Supplemental Figure 9C). Instead, development of resistance to SPA was associated with decreased expression of AR mRNA and protein, decreased AR activity assessed by decreased *KLK3* (encodes PSA) and PSA expression, and loss of suppression of *MYC* (Figure 5, C–F). The AR promoter had reduced accessibility as early as 5 days of SPA, which persisted at 26 days (Figure 5G), consistent with prior reports indicating that ligand-bound AR exhibits negative autoregulation at the level of *AR* gene transcription (31). MYC target gene sets were globally reactivated following development of resistance to SPA (Supplemental Figure 9D), and *MYC* reexpression was associated with reexpression of *PCAT1* and *PVT1* (Supplemental Figure 10A) and reorganization of 8q24 SE accessibility (Supplemental Figure 10B). Dual inhibition of MYC by SPA and the bromodomain inhibitor JQ1 resulted in greater suppression of *MYC* mRNA expression and a longer duration of growth arrest of LNCaP cells than was seen with treatment with SPA alone (Supplemental Figure 11, A and B). This suggests that loss of suppression of MYC was driving acquired resistance to SPA. To determine whether downregulation of AR was driving the loss of MYC suppression and development of acquired resistance, we constitutively expressed AR in LNCaP and LN95 cells (Figure 5H and Supplemental Figure 12A). LNCaP-AR and LN95-AR cells exhibited enhanced suppression of MYC by SPA (Figure 5H and Supplemental Figure 12A), followed by extensive vacuolization (Supplemental Figure 13) and cell death, not resistance (Figure 5I and Supplemental Figure 12B). This indicates that downregulation of AR is a mechanism of acquired resistance to SPA in vitro.

**Figure 6:**
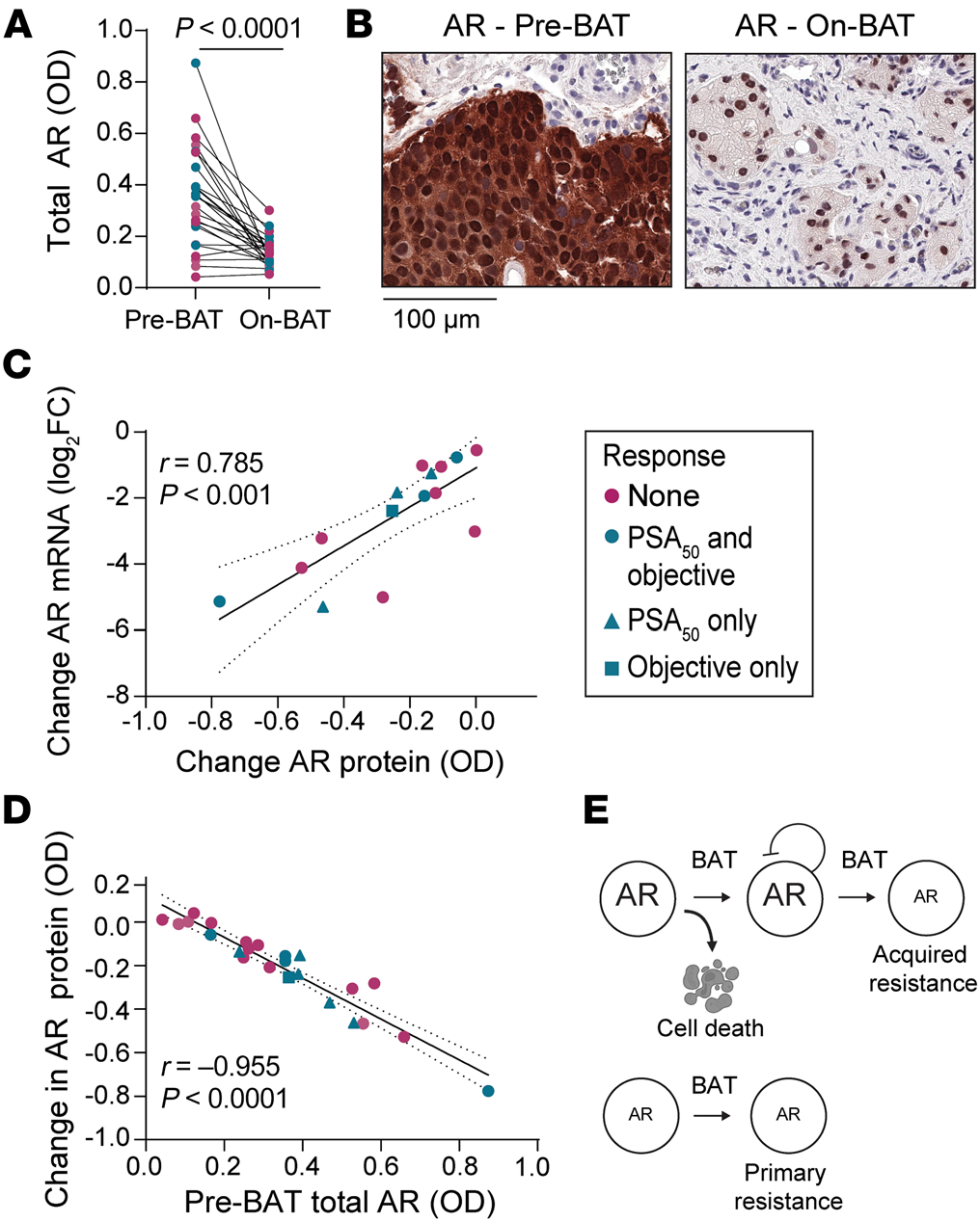
BAT downregulates AR. (**A**) Total AR OD by image analysis in paired tumor biopsies (*n* = 24) color-coded by response. *P* value determined by paired 2-tailed *t* test. (**B**) Example of IHC for AR (1:10,000 antibody dilution) in paired biopsy samples from a responding patient. (**C**) Correlation of *AR* RNA change from C1D1 to C4D1 with AR protein change from C1D1 to C4D1 (*n* = 15). *r* and *P* values determined by Pearson’s correlation calculation. (**D**) Correlation of AR protein change with preBAT total AR OD. *r* and *P* values determined by Pearson’s correlation calculation. (**E**) Schematic model of primary and acquired resistance to BAT.

In the patient samples, we saw that BAT induced strong downregulation of AR protein in most patients (Figure 6, A and B). The change in AR protein levels correlated with the change in *AR* mRNA on BAT (Figure 6C), suggesting that ligand-bound AR inhibits AR gene transcription in patients, as previously described in vitro (31). Higher preBAT AR predicted a greater decrease in AR by BAT (Figure 6D). This might be explained by a threshold effect; effectively, BAT decreases AR to a threshold minimum level below which AR is not further suppressed by BAT. These data demonstrate that adaptive downregulation of AR occurs in patients and might lead to acquired resistance to BAT over time. This mechanism is consistent with an emerging conceptual idea that acquired resistance to cancer therapy is often driven by plastic (i.e. reversible) cellular alterations, rather than gene mutation (32). Overall, these data suggest that low AR expression and activity is a mechanism of primary and acquired resistance to BAT (Figure 6E).

### AR inhibition resensitizes prostate cancer to SPA.

**Figure 7:**
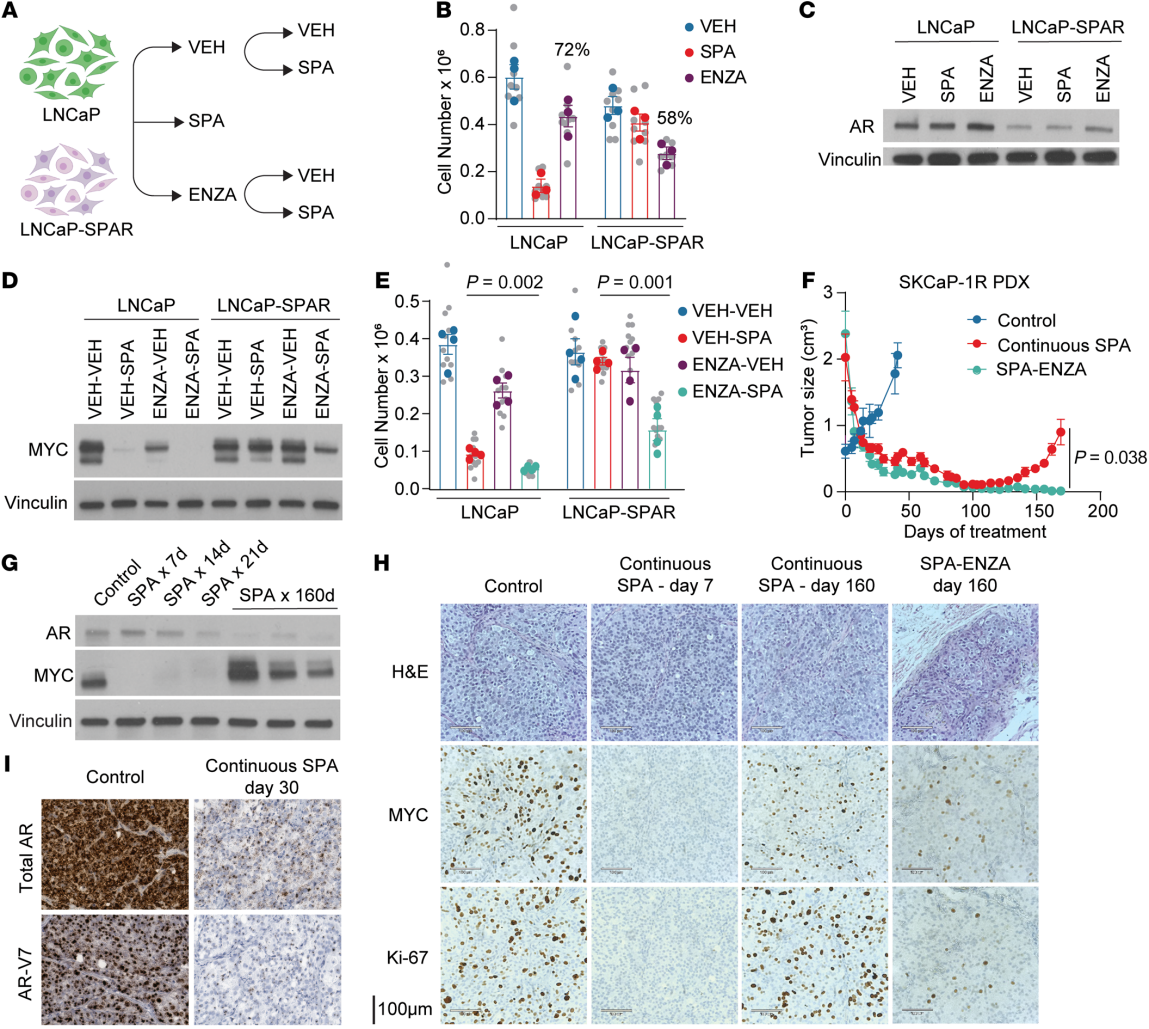
Acquired resistance to SPA can be overcome by alternating between AR activation and inhibition. (**A**) Experimental design schematic. LNCaP-SPAR are LNCaP with acquired resistance to SPA; VEH, vehicle; ENZA, Enzalutamide (**B**) Viable cell number of LNCaP and LNCaP-SPAR cells following treatment with VEH, SPA, or ENZA for 5 days (*n* = 3 independent experiments). Biological replicates indicated in gray with mean of each independent experiment in color. Percent of VEH is indicated for ENZA-treated cells. (**C**) AR protein expression by Western blot of cells treated as indicated in **A**. Representative blot of *n* = 3 independent experiments. (**D**) MYC protein expression by Western blot of LNCaP and LNCaP-SPAR cells following treatment with VEH or ENZA for 5 days followed by VEH or SPA for 5 days. Representative blot of *n* = 3 independent experiments. (**E**) Viable cell number of cells treated as per **D** (*n* = 4 independent experiments). *P* values determined by unpaired 2-tailed *t* test. Biological replicates indicated in gray with mean of each independent experiment in color. (**F**) Tumor size of SKCaP-1R PDX following no treatment (Control; *n* = 4 mice), continuous testosterone (SPA; *n* = 4 mice), or SPA alternating with enzalutamide every 3 weeks (SPA-ENZA; *n* = 3 mice). *P* value determined by unpaired 2-tailed *t* test comparing final measurements. (**G**) AR and MYC protein expression by Western blot of SKCaP-1R untreated (control) or treated with SPA. (**H**) H&E staining and IHC for MYC and Ki-67 of SKCaP-1R following no treatment (Control), continuous SPA for 7 or 160 days, or SPA-ENZA for 160 days. Representative photograph of *n* = 3 mice per group. (**I**) RNA in situ hybridization for *AR* and *AR-V7* in tumors of SKCaP-1R untreated (control) or treated with SPA for 30 days. Representative photograph of *n* = 3 mice per group. For Western blots, vinculin used as a loading control.

We have previously reported that patients whose cancer has progressed while on BAT appear to have enhanced clinical responses to subsequent AR inhibition (6–9). For example, in the TRANSFORMER study, patients with mCRPC who had not received prior BAT exhibited a PSA_50_ response rate of 25% and median response duration of 3.8 months while on the AR inhibitor enzalutamide, while patients who had progressed on BAT exhibited a PSA_50_ response rate of 78% and median duration of response of 10.9 months while on enzalutamide (9). Similarly, LNCaP-SPAR cells were more growth-inhibited by enzalutamide compared to parental LNCaP (Figure 7, A and B). This may be due to a reduction in AR abundance due to prior treatment with SPA (Figure 5F), thereby sensitizing cells to AR inhibition. Notably, enzalutamide treatment resulted in adaptive upregulation of AR in both cell lines (Figure 7C) and enhanced downregulation of MYC (Figure 7D) and growth inhibition by subsequent treatment with SPA (Figure 7E). This indicates that acquired resistance to SPA can be overcome by the use of AR inhibitors like enzalutamide to induce adaptive upregulation of AR.

While BAT was originally designed to cycle testosterone levels with the intention of minimizing adaptation to high or low levels of androgens, these data suggest that there may be clinical benefit to more extreme oscillation of AR activity by alternating the use of SPA with an AR inhibitor. To test this therapeutic strategy, we used a patient-derived xenograft (PDX) model derived from a metastasis of a patient with CRPC and adapted to grow in a castrated mouse (SkCaP-1R) (33). This PDX, which expresses high AR and the AR splice-variant AR-V7 and is resistant to the second generation androgen signaling inhibitors abiraterone and enzalutamide (33), initially regressed in response to SPA, but acquired resistance after about 5 months (Figure 7F). Regression was associated with an almost complete loss of MYC expression, while acquired resistance was associated with a decrease in AR and return of MYC expression (Figure 7, G and H). SPA suppressed mRNA expression of *AR* and *AR-V7* as early as 21–30 days (Figure 7, G–I). Notably, when SPA was alternated every 21 days with enzalutamide (SPA-ENZA), this PDX did not acquire resistance after 160 days of observation (Figure 7F). Subcutaneous tissues from the animals that received SPA-ENZA for 160 days were analyzed histologically and nests of cancer cells were observed, but these cells lacked significant staining for the proliferation marker Ki-67 (Figure 7H). These data suggest that repeat cycling of SPA and AR inhibition may prevent the development of acquired resistance and lead to more durable growth inhibition of prostate adenocarcinoma.

## Discussion

Previous clinical studies have demonstrated that BAT can be an effective therapy for some patients with mCRPC. This study assessed the molecular mechanisms that drive the efficacy of BAT. Our results indicate that high prostate cancer AR activity is required for tumor regression by BAT, which occurs in part through downregulation of MYC. High AR expression was required for downregulation of MYC and growth inhibition by SPA in vitro, as knock down of AR in SPA-sensitive LNCaP cells rescued MYC downregulation and cell cycle arrest following treatment with SPA. Knock-down of AR in these cells achieved AR levels comparable to LAPC4 and 22RV1 cell lines, which exhibit primary resistance to SPA. In patient samples, high AR activity was similarly required for anti-tumor activity of BAT, as there was no patient with a low ARA_MW_ score (defined by our cut-off of less than 0.6) who exhibited significant downregulation of MYC and/or clinical evidence of tumor regression by BAT. Moreover, the prevalence of this biomarker — an ARA_MW_ score greater than 0.6 — among patients with mCRPC in the SU2C/PCF data accurately estimated prior assessments of BAT efficacy in larger clinical trials. If the ARA_MW_ score biomarker is required for a PSA_50_ response to BAT, and the PSA_50_ response rate among biomarker-positive patients is 70%, then the predicted PSA response rate to BAT among patients with mCRPC is 25.6% (i.e., 36.5% × 70%). This is consistent with the measured PSA_50_ BAT-response rate of 24.3% among 173 patients with mCRPC across 2 clinical trials (7–9), and it provides further plausibility that the ARA_MW_ score can predict response to BAT. Together, our results suggest that an ARA_MW_ score greater than 0.6 is required for clinical benefit from BAT, and this gene expression score could function as a biomarker for patient selection if validated. A limitation to the use of this biomarker is that it requires fresh tumor tissue, which can be difficult to obtain in some patients. Therefore, investigation into alternative surrogate biomarkers using molecular imaging or circulating tumor markers may be clinically useful.

We also observed that primary resistance to SPA can be overcome by forced overexpression of AR in LAPC4 and 22Rv1 cells in vitro. Similarly, Litvinov et al. previously demonstrated that forced overexpression of AR in PC3 cells confers sensitivity to growth inhibition by SPA (34). It should be noted, however, that high AR activity may not be universally sufficient to confer sensitivity of prostate cancer to SPA, as DU145 cells transduced to express high AR remain resistant to growth inhibition by SPA, even with high AR overexpression (34). We observed that 3 of 15 patients in our cohort had ARA_MW_ scores greater than 0.6 but did not obtain a PSA_50_ or objective response to BAT. While these 3 patients did not meet guideline definitions for a clinical response to BAT, 2 of these patients experienced prolonged overall survival following BAT, most similar to patients who did exhibit a clinical response. This suggests that biochemical and objective responses may not predict overall benefit from BAT for some patients. Nonetheless, it seems that while high AR activity is required for prostate cancer growth inhibition by BAT, it may not be sufficient, and future studies should assess additional factors that are required to confer sensitivity to BAT.

A subtlety of our findings is that tumor AR activity, but not AR abundance, predicted clinical outcomes for patients on BAT. We developed a quantitative method using IHC to measure AR abundance in tissue. AR protein abundance was highly correlated with *AR* mRNA abundance by RNA-Seq data, but not with AR activity. This supports prior work showing that factors beyond AR protein abundance determine AR activity (28, 35). These factors may include AR gene mutations that alter protein activity and/or abundance of coregulators that can amplify or diminish AR function. A better understanding of the regulation of AR activity may allow for the development of strategies to boost and sustain AR activity in order to enhance sensitivity to BAT.

A key question to understanding BAT is: what are the molecular events that result in regression of tumors with high AR activity? Our results suggest that SPA/BAT can significantly downregulate MYC expression, and this occurs only in prostate cancer with high AR activity. This is in accordance with prior reports that demonstrate the ability of SPA to downregulate MYC in prostate cancer cell lines (1, 13–15), but is the first evidence that this phenomenon occurs in patients. Guo et al. recently showed that SPA can result in repression of MYC transcription in prostate cancer cell lines with high AR expression by altering SE activity on 8q24 (13). In support of this mechanism, we show that in patient samples, the change in MYC expression following BAT directly correlates with the change in expression of genes coregulated by SEs on 8q24. Interestingly, Guo et al. also demonstrate that repression of MYC by SPA occurs through a process independent of AR DNA binding (13). This may suggest that highly active ligand-bound AR can bind and sequester, or squelch, factors required for transcription of MYC. The identification of these factors that become limiting for MYC transcription following BAT may lead to the testing of combination therapy strategies to concurrently target these factors with BAT with a goal of achieving greater therapeutic efficacy. Notably, we find that forced constitutive expression of MYC in SPA-sensitive cell lines only achieves a partial rescue of growth inhibition by SPA. This suggests that downregulation of MYC contributes to, but is not the sole mechanism of, growth inhibition by SPA/BAT. We anticipate that MYC-independent maladaptive effects of SPA may also contribute to growth inhibition, such as AR inhibition of DNA relicensing during mitosis (16), AR-mediated DNA damage (12, 17), and induction of ferroptosis and immunogenic cell death (18).

Acquired resistance is a significant limitation to the use of BAT. We show that acquired resistance to SPA can be driven by downregulation of AR in vitro. BAT induced downregulation of AR mRNA and protein in most patients in our study, consistent with prior reports indicating that ligand-bound AR exhibits negative autoregulation at the level of *AR* gene transcription (31). We found that acquired resistance could be prevented in the SPA-sensitive cell line LNCaP through constitutive AR expression, which is not subject to negative autoregulation. Therefore, methods to prevent AR negative autoregulation have the potential to prevent acquired resistance to BAT. To our knowledge, no such methods currently exist, however, AR-axis inhibitors are widely known to induce adaptive upregulation of AR in patients with CRPC (10, 11). Thus, we tested sequential treatment of prostate cancer models with SPA followed by the AR inhibitor enzalutamide. Our results indicate that acquired resistance to SPA can be overcome by treatment with enzalutamide, which induced upregulation of AR and increased susceptibility to SPA.

This study indicates that prostate cancer engages in classical endocrine negative feedback loops to titrate AR to match the abundance of ligand. Our results suggest that these feedback loops generate vulnerabilities that researchers might be exploited therapeutically. To test this clinically, we have initiated a clinical trial in which patients are repeatedly switched from BAT to enzalutamide to BAT, entitled, “Sequential Testosterone and enzalutamide Prevents Unfavorable Progression (STEP-UP)” (NCT04363164). By shifting the treatment strategy to anticipate resistance, this dynamic protocol is an attempt to use game theory (36) to minimize resistance and maximize physician control of prostate cancer growth.

Some of the limitations of our study are that it included a relatively small number of patients and was restricted to patients and models of prostate adenocarcinoma. While some have postulated that BAT may reverse transdifferentiation of prostate adenocarcinoma to neuroendocrine prostate cancer, which can occur as a mechanism of resistance to AR-axis inhibitors (37), we did not assess this possibility in the current study. We anticipate that prostate cancer of diverse histology with low AR activity will not be growth-inhibited by SPA/BAT. However, beyond a direct inhibition of cellular proliferation, it is possible that BAT can alter other cellular behaviors that promote cancer fitness, such as invasion, metastasis, and evasion of the immune system, which may or may not be dependent on cancer cell AR activity. Our study did not directly measure these other processes, although they may be indirectly captured by patient overall survival, which implies that overall benefit from BAT is mainly restricted to tumors with high AR activity. Future studies should assess these effects directly to more fully understand the potential utility of BAT.

Overall, this study may enable rational use of BAT for treatment of mCRPC. We identified a subgroup of patients most likely to benefit from BAT and a strategy to limit acquired resistance to this therapy, which should be validated in clinical trials. Moreover, we believe that this work promotes a new paradigm for treatment of advanced prostate cancer. While the status quo, as it pertains to treatment of advanced prostate cancer, is persistent and potent AR inhibition, this work provides rationale to alternate between AR inhibition and activation with BAT to prolong the lives of patients with this disease.

## Methods

### Cell culture and reagents.

LNCaP and VCaP cell lines were obtained from ATCC. LAPC4 and 22Rv1 cell lines were a gift from J Isaacs (Johns Hopkins, Baltimore). LNCaP, LAPC4, and 22Rv1 were grown in RPMI 1640 (Thermo Fisher Scientific) supplemented with 10% FBS (Corning), sodium lactate 1.6 mM, sodium pyruvate 0.5 mM, L-alanine 0.43 mM, and 1% pen-strep (Thermo Fisher Scientific). VCaP was grown in DMEM (ATCC) supplemented with 10% FBS (Corning), and 1% pen-strep (Thermo Fisher Scientific). Full-length AR cDNA was cloned into the BAMH1/Sal1 site of pLenti-CMV-GFP-Puro (Addgene; 17448, E Campeau and P Kaufman laboratories), and empty vector control was generated through excision of GFP of this plasmid. These vectors, along with pCDH-puro-cMyc vector (Addgene; 46970, J Wang laboratory), pCDH-EF1-FHC empty vector control (Addgene, 64874, R Wood laboratory), piSMARTvector-PGK-TurboGFP-TRE3G-shAR vectors (Horizon; V3IHSPGG_8216343 and V3IHSPGG_7292640) were transfected into 293T cells (ATCC) along with pMD2.G (Addgene) and psPAX2 (Addgene) packaging vectors using lipofectamine (Invitrogen) to produce AR-puro, cMyc-puro, control-puro, and Tet-On-shAR-puro lentivirus particles. 2 days after transduction with the indicated virus, vector-expressing cells were selected with puromycin 1 μg/mL for 72 hours. To constitutively express MYC in LAPC4-AR cells, LAPC4-AR cells were transfected with pCDNA3-HA-HA-human MYC vector (Addgene; 74164, M Roussel laboratory) using lipofectamine (Invitrogen) then selected for stable expression with G418 (Sigma-Aldrich) for 7 days. Cells were maintained at 37°C in 5% CO_2_. The cells regularly tested negative for mycoplasma contamination using MycSensor PCR Assay kit (Agilent Technologies). R1881 was obtained from Sigma-Aldrich, enzalutamide from SelleckChem, and testosterone cypionate from Steraloids.

### Proliferation, cell viability, clonogenic survival, and cell cycle analyses of cell lines.

For proliferation and cell viability assays, cells were plated in triplicate on 6-well (0.1 × 10^6^ cells/well) or 12-well (0.05 × 10^6^ cells/well) plates and incubated with R1881, enzalutamide, or vehicle control for the indicated duration. Cells were counted using a hemocytometer with viability assessed by trypan blue exclusion. For clonogenic survival assessment, cells were plated on 6-well plates at low density (1,500 cells/well) in 1 mL fresh media and 250 μL conditioned media obtained from a confluent flask of the parental cell line. Colonies were stained with crystal violet (Sigma-Aldrich; 0.5% in 20% methanol) after 10–20 days. For cell cycle analysis, cell pellets were resuspended in cold 70% ethanol for a minimum of overnight, then subsequently washed with PBS and resuspended in 50 μg/mL propidium iodide (Sigma-Aldrich) and 100 μg/mL RNAse (Sigma-Aldrich) and run on the BD FACSCelesta flow cytometer with analysis using FlowJo software 10.4.2.

### Western blot analyses.

Cells or tissues were lysed with 1 × denaturing lysis buffer (Cell Signaling Technology) containing protease and phosphatase inhibitors (Roche). Protein concentration was determined using the Pierce BCA Protein Assay kit (Thermo Fisher Scientific), and 5–20 μg of lysate was resolved on a SDS-PAGE gel and transferred to a nitrocellulose membrane. Membranes were blocked with 5% milk for 1 hour, then incubated with primary antibody overnight. Primary antibodies used were anti-cMYC (Abcam, clone Y69, 1:1,000 dilution), anti-AR (Santa Cruz Biotechnology, clone sc7305, 1:1,000 dilution), anti-PSA (Agilent Technologies, clone A0562, 1:1,000 dilution), anti-vinculin (Sigma-Aldrich, clone V284, 1:2,000 dilution). Anti-rabbit IgG HRP-linked antibody (Cell Signaling Technology, clone 7074S, 1:5,000 dilution) and anti-mouse IgG HRP-linked antibody (Cell Signaling Technology, clone 7076S, 1:5,000 dilution) were used as secondary antibodies. Please refer to Supplemental Figures 14–19 to view full uncut Western blots.

### Quantitative real-time PCR.

RNA was extracted using the RNeasy kit (Qiagen) and cDNA generated using the high-capacity cDNA reverse transcription kit (Thermo Fisher Scientific). Real-time PCR was performed in triplicate using 500 μg cDNA, 10 μL TaqMan Gene Expression Master Mix (Thermo Fisher Scientific), and 1 μL 20 × TaqMan Gene Expression Assay probe mix for *MYC* (Hs00153408_m1), *AR* (Hs00907244_m1), *KLK3* (Hs02576345_m1), and *ACTB* (Hs01060665_g1; Thermo Fisher Scientific) on an ABI7500 Real-Time PCR System (Thermo Fisher Scientific). Relative gene expression was determined by ΔΔCT.

### Clinical trial design and procedures.

The COMbination of BAT and Nivolumab (COMBAT-CRPC; NCT03554317) clinical trial was a single-arm, multicenter, open-label phase II study of BAT in combination with the anti-PD1 agent nivolumab for patients with CRPC that had progressed on at least 1 novel AR-target therapy. The inclusion and exclusion criteria and prespecified study end points were previously described (38). Patients were required to have soft tissue metastases amenable to biopsy to participate. Patients were treated with 3 cycles of BAT — testosterone cypionate 400 mg intramuscular every 28 days — followed by concurrent BAT and 480 mg nivolumab intravenously every 28 days until progression. Paired core needle tumor biopsies were performed prior to treatment and after 3 cycles of BAT monotherapy. Response was assessed with PSA at each cycle, and chest, abdomen, and pelvis CT scans and technetium-99 bone scan every 3 cycles.

### Mouse models and tumor studies.

Male NSG mice aged 8–12 weeks were obtained from the Sidney Kimmel Comprehensive Cancer Center Animal Core Facility and surgically castrated. SKCaP PDX tissue was minced, mixed with Matrigel (BD Biosciences) and implanted subcutaneously on the flank. Testosterone was administered by implantation of a slow-release subcutaneous pellet in the opposite flank. Pellets were assembled using 25 mm sections of Silastic Laboratory tubing, filled with 30 mg testosterone cypionate (Steraloids), sealed on both ends with Silastic Medical Adhesive Type A, then sterilized. Enzalutamide (SelleckChem) was administered by oral gavage 10 mg/kg/day in 200 μL 1% carboxymethyl cellulose, 0.1% Tween-80, 5% DMSO. Tumors were measured twice weekly using microcalipers, and tumor volume was calculated using the following formula: 0.5236 × L × W × H. At study completion, the mice were euthanized and the tumors were extracted. Tumors were flash frozen for subsequent lysis for immunoblot analyses and formalin-fixed for subsequent IHC analyses. All mice were housed in the Johns Hopkins animal facility.

### Data availability.

The sequencing data described have been deposited in NCBI’s Gene Expression Omnibus and are accessible through GEO Series accession number GSE213444.

### Statistics.

Results are displayed as mean ± SD, unless otherwise indicated. Statistical comparison between groups was performed using a 2-tailed Student’s *t* test using GraphPad Prism v8.0 software. Statistical comparisons of proportions were performed using a Chi-squared test. Statistical comparisons of time-to-event data were performed using Kaplan-Meier analysis with log-rank test using GraphPad Prism v8.0. Analysis of correlation was performed using Pearson’s correlation calculation. *P* values less than 0.05 were considered statistically significant.

### Study approval.

The COMbination of BAT and Nivolumab (COMBAT-CRPC; NCT03554317) clinical trial was approved by the IRBs at Johns Hopkins, Dana Farber, and University of San Francisco, and all accrued patients provided written informed consent. For animal studies, we have complied with all relevant ethical regulations in accordance and approved by the Johns Hopkins IACUC.

## Author contributions

LAS, ESA, AMD, JTI, MCM, and SRD conceptualized the study. LAS, RK, DES, ESA, AMD, JTI, MCM, and SRD developed the methodology. LAS, RK, DES, EAT, DMR, SLD, LA, YY, CGA, JLH, TJ, KAB, JNE, JM, AG, and AS performed the experiments. LAS and DES visualized the study. ESA, SKK, and SRD acquired funding for the project. SY, JL, WNB, SKK, ESA, AMD, JTI, MCM, SRD supervised the study. LAS wrote the original draft of the manuscript. All authors were responsible for reviewing and editing the manuscript. The order of co–first authors was determined based on their contributions to the manuscript.

## Supplementary Material

Supplemental data

## Version 1 10/04/2022

In-Press Preview

## Version 2 12/01/2022

Electronic publication
