# Supplementary material for: Androgen receptor activity in prostate cancer dictates efficacy of bipolar androgen therapy through MYC
Source: J Clin Invest. 2022 Dec 1;132(23):e162396. doi: 10.1172/JCI162396 (PMC9711876; doi:10.1172/JCI162396)
Supplement: Supplemental data [file jci-132-162396-s086.pdf]

Supplemental Material

Prostate cancer androgen receptor activity dictates efficacy of bipolar androgen therapy through MYC

Laura A Sena, Rajendra Kumar, David E Sanin, Elizabeth A Thompson, D Marc Rosen, Susan L Dalrymple, Lizamma Antony, Yuhan Yang, Carolina Gomes-Alexandre, Jessica L Hicks, Tracy Jones, Kiara A Bowers, Jillian N Eskra, Jennifer Meyers, Anuj Gupta, Alyza Skaist, Srinivasan Yegnasubramanian, Jun Luo, W Nathaniel Brennen, Sushant K Kachhap, Emmanuel S Antonarakis, Angelo M De Marzo, John T Isaacs, Mark C Markowski, Samuel R Denmeade

Supplemental Methods

Chromogenic immunohistochemistry (IHC) for AR.
Quantitative image analysis for AR protein.
Immunohistochemistry (IHC) for MYC and Ki67 proteins.
Frozen biopsy specimens for laser capture microdissection (LCM) for bulk RNAseq analysis.
RNA sequencing.
ARA_{MW} score calculation.
ATAC sequencing.
RNA in situ hybridization (RISH).

Supplemental References (for Methods)

Supplemental Tables

Table S1. Patient characteristics.

Supplemental Figures

Figure S1. LNCaP and VCaP are SPA-sensitive prostate cancer cell lines, while LAPC4 and 22Rv1 are SPA-resistant cell lines.
Figure S2. AR knock-down rescues clonogenic survival of LNCaP treated with SPA.
Figure S3. Downregulation of MYC is specific to growth inhibition by SPA.
Figure S4. Total AR protein abundance correlates with nuclear AR, cytoplasmic AR, and AR mRNA in patient biopsies.
Figure S5. ARA_{MW} score integrates expression of a panel of AR target genes and does not correlate with AR protein expression.
Figure S6. Characterization of the ARA_{MW} score.
Figure S7. Unbiased analyses of RNA sequencing of paired patient biopsies.
Figure S8. Homologous recombination repair (HRR) gene alterations do not predict response.
Figure S9. Cells with acquired resistance to SPA revert to a pre-treatment phenotype but maintain AR activation.
Figure S10. Acquired resistance to SPA is associated with an alteration in super-enhancer activity on 8q24.
Figure S11. Dual inhibition of MYC by SPA and JQ1 prolongs growth inhibition of LNCaP cells.
Figure S12. Constitutive expression of AR in LN95 cells reduces acquired resistance to SPA.
Figure S13. SPA results in extensive vacuolization in LNCaP cells with constitutively high AR expression.

Uncut Western Blots

Figure S14. Full blots for Figure 1.
Figure S15. Full blots for Figure 2.
Figure S16. Full blots for Figure 5.
Figure S17. Full blots for Figure 7.
Figure S18. Full blots for Figure S3B.
Figure S19. Full blots for Figure S12A.

Supplemental Methods

Chromogenic Immunohistochemistry (IHC) for AR

Tissue sections were baked on a 60℃ hotplate for 10 min. and then deparaffinized in xylenes. Tissues were rehydrated in graded alcohol solutions and distilled water (diH_2O). Slides were then rinsed in a solution of diH_2O with 0.1% Tween and placed in a citrate bucket wash for 1 min. Tissue sections were steamed in Target Retrieval Solution (Dako; S1699, Santa Clara, CA), washed in Tris Buffered Saline with Tween (TBST; Sigma), and subjected to a Dual Endogenous Enzyme Block (Dako; S2003). The tissues were incubated with the primary antibody (conditions varied for each antibody) and, rinsed with TBST, and incubated with the secondary antibody, PowerVision Poly-HRP anti-Rabbit IgG (Leica, Deer Park, IL), for 30 min. at RT. Tissues were rinsed with TBST and then aminoethyl carbazole (Immpact AEC; Vector Labs; SK-4205, Burlingame, CA) was applied for 20 min. at RT. Slides were washed with TBST and counterstained with hematoxylin (Mayers; Dako; S3309; diluted 1:4). Slides were then coverslipped with VectaMount AQ Aqueous Mounting Medium (Vector Laboratories; H-5501) and scanned on a Ventana DP200 Digital Whole Slide Scanner (Roche Diagnostics, Rotkreuz, Switzerland) using a 40X microscope objective. Coverslips were removed with diH_2O and AEC was dissolved with graded levels of alcohol. Slides were then steamed and incubated with an SDS-2ME solution as previously described (*39*). Slides were placed in TBST for 5 min. and then steamed, after which the dual endogenous block was reapplied and the tissue was incubated with the next antibody. This process was repeated until each tissue had been iteratively stained with the following antibodies:

Iteration 1- Anti-AR (Cell Signaling, Rabbit monoclonal, Clone D6F1I, 1:10,000) overnight at 4 degrees Celsius.
Iteration 2 – Anti-AR (Cell Signaling, Rabbit monoclonal, Clone D6F1I, 1:5,000) overnight at 4 degrees Celsius.
Iteration 3 – Anti-CK8 (Abcam, Rabbit monoclonal, Clone EP1628Y, 1:3,000) 45 minutes at room temperature.

As a control, after primary antibody removal, for each of the antibody staining rounds in a number of experiments we performed staining by leaving out the primary antibody and performing a round of staining with the secondary antibody only. These experiments showed a complete absence of signals indicating a complete antibody removal.

Quantitative Image Analysis for AR Protein

To quantify AR separately in the nucleus, cytoplasm, and whole cell separately, we developed and performed an iterative IHC assay using both AR and keratin 8, the latter which was used to help delineate tumor epithelium in the metatstic lesions . Wholeslide scans of each staining round for AR and CK8 were imported into HALO. Each scan was then subjected to color deconvolution to generate a separate hematoxylin and IHC channel, and these whole slide deconvoluted images were then registered with one another into a fused multiplex pseudo-colored whole slide image as described(*40*). A patient-specific Random Forest classifier was manually trained to classify all regions of each CK8-stained tissue into one of three categories: tumor, non-neoplastic tissue, and background. Annotations created from this classifier were then mapped onto the scans of the AR-stained tissues, using HALO's "Classify Registered" feature. In the event that the scan of the CK8-stained tissue could not be successfully used for tumor classification (low CK8 expression, degraded tissue or imprecise scan registration), a patient-specific tumor classifier was trained on the scan of the AR-stained tissue. If the AR signals at a dilution of 1:10,000 was too weak for classifier training, scans of the tissues stained for AR at 1:5,000 were utilized to train the classifier. AR positivity thresholds were determined using the real-time tuning window. Quantitative image analysis was performed using the MultiPlex module on HALO 3.2 (Indica Labs). The MultiPlex analysis was restricted to tumor positive regions (using the CK8-trained classifier) and provided several measurements for each tumor-positive cell within each AR-stained tissue, including nuclear AR optical density (OD), cytoplasmic AR OD, nuclear area (μm^2), cytoplasmic area (μm^2), and cell area (μm^2). Whole cell average AR ODs were determined by calculating an integrated cell OD for each cell and dividing that value by each cell's respective area.

$$Average\ Cell\ OD = \frac{(Nuclear\ AR\ OD\ *\ Nuclear\ Area) + (Cytoplasmic\ AR\ OD\ *\ Cytoplasmic\ Area)}{Cell\ Area}$$

Immunohistochemistry (IHC) for MYC and Ki67 Proteins

IHC on patient biopsy samples for MYC and Ki67 was performed as previously described (*20*, *41*). IHC on SKCaP PDX tissue was performed by the Sidney Kimmel Comprehensive Cancer Center (SKCCC) IHC Core facility. Primary antibodies were anti-MYC (Epitomics, Clone Y69, 1:200) and anti-Ki67 (AbCam, Clone SP6, 1:200), and tissues were stained with the Ventana Discovery Ultra Autostainer, using the Discovery anti-HQ HRP kit.
Quantitative Image Analysis for MYC and Ki67 Proteins

For quantitative image analysis, whole IHC stained biopsy slides were scanned using a 40X objective on a Ventana DP200 slide scanner. Whole slide scans were imported into HALO 3.1 image analysis platform. The nuclear staining for both MYC and Ki67 were analyzed using the cytonuclear module v 1.6. Tumor regions were delineated by a pathologist (CG-A) using manual annotations, guided by adjacent H&E and androgen receptor stained slides. Positively stained (DAB staining above minimum intensity threshold) for MYC and Ki67 cells were binned into 3 intensity levels by the software. We calculated the percentage of positively stained cells for both Ki67 and MYC proteins, as well as an H-SCORE for MYC using the intensity levels with the following formula (percentage of intensity level 3 cells x 3)+(percentage of intensity level 2 cells x 2)+ (percentage of intensity level 1 cells x 1) to give a maximum possible score of 300. Percentage positively stained cells were validated on a number of samples by manual counting.

Frozen biopsy specimens for laser capture microdissection (LCM) for bulk RNAseq analysis

Frozen sections were cut onto PEN (polyethylene naphthalate) slides (Leica), stained with hematoxylin and LCM was performed at the SKCCC Core facility using a Leica LMC7000 laser capture microdissection system in the SKCCC Cell Imaging Core. Laser captured tissues from regions highly enriched for tumor cells were collected by manual annotations of regions of interest that outlined tumor nests. RNA was isolated using the ALLPREP RNA/DNA extraction protocol (Qiagen Cat. No. 802804). RNA amounts were measured using a Qubit fluorometer (Invitrogen) and quality were measured using an Agilent Bioanalyzer 2100 with the Pico Chips Kit.

RNA sequencing

For RNA sequencing of patient biopsy samples, purified RNA was provided to the SKCCC Experimental and Computational Genomics Core to carry out their low-input RNA-seq workflow as described previously with some modifications (*42*). Briefly, quality of total RNA was measured by the Agilent Bioanlayzer to determine RNA integrity (RIN). Samples with starting input between 100pg-100ng of total RNA and RIN > 7.0 were considered to have sufficient quality to proceed to construction of whole transcriptome sample-barcoded libraries using the Ovation RNA-Seq System V2 according to the manufacturer's protocols (Nugen). Quantification of the libraries was performed by qPCR or by the Agilent Bioanalyzer and equimolar concentrations of each library were pooled together, clustered and sequenced on an Illumina Novaseq 6000 platform, with paired end sequencing. The resulting reads were aligned the human reference genome build hg38 using RSEM (*43*) with the STAR aligner (*44*) to obtain posterior mean estimate transcripts per million (pmeTPM) as a measure of gene expression. pmeTPM values for each gene were normalized using upper quantile normalization across samples and log2 transformed. For RNA sequencing of cell lines, purified RNA was sent to Admera Health who performed library preparation with polyA selection, sequencing on the Illumina HiSeq 2500 with read length configuration of 2x150bp with 60 million reads per sample, and read mapping to GRCM38. Sequenced libraries were processed with deepTools (*45*), using STAR (*44*), for trimming and mapping, and featureCounts (*46*) to quantify mapped reads. Raw mapped reads were processed in R (Lucent Technologies) with DESeq2 (*47*) to generate normalized read counts to visualize and cluster as heatmaps using pheatmap [53] and determine differentially expressed genes with greater than 2-fold change and lower than 0.01 adjusted p value. Gene set enrichment analysis was performed using fGSEA (*48*).

ARA_{MW} Score Calculation

We calculated the AR activity score using Mann-Whitney ranking (ARA_{MW}) by adapting a previously reported approach (*49*). First, genes were selected based on previous reports (*50*–*52*) and examined for co-linearity

based on the pearson correlation coefficient and testing for its significance using the cor and cor.test functions in R, respectively. Having observed no correlation between most genes, we selected 10 genes (*KLK2, KLK3, FKBP5, STEAP1, STEAP2, PPAP2A, RAB3B, ACSL3, NKX3-1, TMPRSS2*) and applied the adapted gene scoring method to RNAseq results from COMBAT biopsies, and to polyA captured RNAseq results from 266 biopsies from participants in the SU2C/PCF study (*29*) retrieved from cBioPortal (*53*). Briefly, we used the log transformed quantile normalized pmeTPM values and calculated a ranked list of genes for each biopsy to generate a $g\ x\ b$ matrix R of ranked genes (with g genes and b biopsies), considering only the top 1500 highest ranked genes by setting $r_{g,b} = r_{max} + 1$, with $r_{max} = 1500$. The ARA_{MW} score was then calculated for the n selected genes as:

$$ARA_{MW} = 1 - \frac{U_b}{n * r_{max}}$$

where U is the Mann-Whitney U statistic calculated as:

$$U_b = \sum_{i=1}^{n} \quad r'_{i,b} - \frac{n(n+1)}{2}$$

and R' is a subset of R including only the n selected genes in the score. In this manner, the score was independent of sequencing depth, normalization strategy and dataset composition, and was primarily based on the relative abundance of selected genes within each studied sample.

Clinical data from 266 participants in the SU2C/PCF was retrieved from cBioPortal and participants classified based on ARA_{MW}. Differences between classified patients for each clinical parameter was examined using a Wilcoxon ranked sum test.

ATAC sequencing

Cells were provided to the Single Cell and Transcriptomics Core at Johns Hopkins University who performed nuclei isolation, library preparation, and sequencing with read length configuration of 2x75 with 50 million reads per sample. Briefly, $5x10^4$ cells were washed in PBS and then lysed in 10 mM Tris-HCl, pH 7.4,10 mM NaCl, 3 mM $MgCl_2$ and 0.1% Igepal CA-630 (all SIGMA). Nuclei were then spun down and then resuspend in 25 µL TD (2x reaction buffer), 2.5 µL TDE1 (Nextera Tn5 Transposase) and 22.5 µL nuclease-free water, incubated for 30 min at 37°C. Purified DNA was PCR-amplified, then cleaned from adaptors. Sequenced libraries were trimmed with Trimmomatic (*54*), mapped using Bowtie2 (*55*), and processed with SAM tools (*56*) to retain open chromatin fragments of less than 150kbp. Coverage files were generated with deepTools (*45*). Open chromatin and differentially regulated peaks were detected with MACS2 (*57*) with a p value $< 1 \times 10^{-7}$ and a q value of less than 0.1 and DiffBind (*58*) using a 2-fold change and 0.01 p value as significance thresholds. Bed files were analyzed with Bedtools (*59*), and visualized alongside coverage files on IGV (*60*). Reference H3K27Ac CHIP-seq on human VCAP data was obtained from the ENCODE project (*61*).

RNA in situ hybridization (RISH)

RISH was performed as previously described (*62*).

Supplemental References (for Methods)

39. Gendusa R, Scalia CR, Buscone S, Cattoretti G. Elution of high-affinity (>10-9 KD) antibodies from tissue sections: Clues to the molecular mechanism and use in sequential immunostaining. *J. Histochem. Cytochem.* 2014;62(7):519–531.
40. Ozbek B et al. Multiplex immunohistochemical phenotyping of T cells in primary prostate cancer. *Prostate* 2022;82(6):706–722.
41. Trabzonlu L et al. Molecular pathology of high-grade prostatic intraepithelial neoplasia: Challenges and opportunities [Internet]. *Cold Spring Harb. Perspect. Med.* 2019;9(4). doi:10.1101/cshperspect.a030403
42. Freeman ZT et al. A conserved intratumoral regulatory T cell signature identifies 4-1BB as a pan-cancer target. *J. Clin. Invest.* 2020;130(3):1405–1416.
43. Li B, Dewey CN. RSEM: accurate transcript quantification from RNA-Seq data with or without a reference genome. *BMC Bioinformatics* 2011;12(1):323.
44. Dobin A et al. STAR: ultrafast universal RNA-seq aligner. *Bioinformatics* 2013;29(1):15–21.
45. Ramírez F et al. deepTools2: a next generation web server for deep-sequencing data analysis. *Nucleic Acids Res.* 2016;44(W1):W160-5.
46. Liao Y, Smyth GK, Shi W. featureCounts: an efficient general purpose program for assigning sequence reads to genomic features. *Bioinformatics* 2014;30(7):923–930.
47. Love MI, Huber W, Anders S. Moderated estimation of fold change and dispersion for RNA-seq data with DESeq2. *Genome Biol.* 2014;15(12):550.
48. Korotkevich G et al. Fast gene set enrichment analysis [Internet]. *bioRxiv* 2016; doi:10.1101/060012
49. Andreatta M, Carmona SJ. UCell: Robust and scalable single-cell gene signature scoring. *Comput. Struct. Biotechnol. J.* 2021;19:3796–3798.
50. Spratt DE et al. Transcriptomic heterogeneity of androgen receptor activity defines a de novo low AR-active subclass in treatment naïve primary prostate cancer. *Clin. Cancer Res.* 2019;25(22):6721–6730.
51. Faisal FA et al. Racial variations in prostate cancer molecular subtypes and androgen receptor signaling reflect anatomic tumor location. *Eur. Urol.* 2016;70(1):14–17.
52. Qiu X et al. MYC drives aggressive prostate cancer by disrupting transcriptional pause release at androgen receptor targets. *Nat. Commun.* 2022;13(1):2559.
53. Cerami E et al. The cBio cancer genomics portal: an open platform for exploring multidimensional cancer genomics data. *Cancer Discov.* 2012;2(5):401–404.
54. Bolger AM, Lohse M, Usadel B. Trimmomatic: a flexible trimmer for Illumina sequence data. *Bioinformatics* 2014;30(15):2114–2120.
55. Langmead B, Salzberg SL. Fast gapped-read alignment with Bowtie 2. *Nat. Methods* 2012;9(4):357–359.
56. Li H et al. The Sequence Alignment/Map format and SAMtools. *Bioinformatics* 2009;25(16):2078–2079.
57. Zhang Y et al. Model-based analysis of ChIP-Seq (MACS). *Genome Biol.* 2008;9(9):R137.
58. Ross-Innes CS et al. Differential oestrogen receptor binding is associated with clinical outcome in breast cancer. *Nature* 2012;481(7381):389–393.
59. Quinlan AR, Hall IM. BEDTools: a flexible suite of utilities for comparing genomic features. *Bioinformatics* 2010;26(6):841–842.
60. Robinson P, Zemo jtel T. Integrative genomics viewer (IGV): Visualizing alignments and variants. In: *Computational Exome and Genome Analysis*. Chapman and Hall/CRC; 2017:233–245
61. Zhang J et al. An integrative ENCODE resource for cancer genomics. *Nat. Commun.* 2020;11(1):3696.
62. Zhu Y et al. Novel junction-specific and quantifiable in situ detection of AR-V7 and its clinical correlates in metastatic castration-resistant prostate cancer. *Eur. Urol.* 2018;73(5):727–735.

Supplemental Tables

Table S1. Patient characteristics

ID	Age (years)	Prior Therapy	Disease volume*	Site of Biopsy	Baseline PSA (ng/ml)	PSA on C4D1 (ng/ml)	Change tumor volume on C4D1 (%)	Response designation
1	63	Abi, Enza, Taxane, Other AR-targeted	High	Retroperitoneal LN	27.8	3.2	-16	R
3	70	Abi, Enza, Taxane	High	Para-aortic LN	166.6	151.7	-50	R
5	66	Enza	High	Paratracheal LN	34.2	70	NA	NR
6	72	Abi, Enza	Low	Axillary LN	147.9	165.8	-1	NR
7	68	Abi, Enza, Taxane, Other AR-targeted	Low	Retrocaval LN	249.4	123.3	-30	R
8	56	Abi, Taxane	Low	Pelvic LN	11.9	24.6	-17	NR
10	69	Abi	Low	Para-aortic LN	18.2	14.6	-15	NR
11	64	Abi, Enza, Taxane	High	Axillary LN	8.0	2	-50	R
12	70	Abi, Enza	High	Retrocrural LN	100.0	45.1	-53	R
13	72	Abi, Enza	High	Retroperineal LN	366.3	20.6	-64	R
18	61	Abi, Enza	High	Liver	51.4	94.6	71	NR
20	72	Enza	High	Liver	50.4	228.1	17	NR
23	86	Abi, Enza, Taxane, other AR-targeted	High	Inguinal LN	79.2	113.80	-14	NR
24	69	Abi	Low	Para-aortic LN	92.9	4.6	-23	R
29	66	Abi, Enza, Taxane	High	Supraclavicular LN	185.2	54.2	-13	R
30	61	Abi, Taxane	Low	Para-aortic LN	16.2	62.1	30	NR
31	68	Abi	Low	Para-aortic LN	29.8	16.7	-13	NR
32	51	Abi, Enza, Taxane		Pre-sacral/common iliac LN	168.4	109.40	-13	NR
35	75	Abi, Enza, Taxane	High	Lung	240.9	516.70	-22	NR
47	57	Abi	Low	Mediastinal LN	5.4	16	0	NR
49	70	Abi, Enza	Low	Para-aortic LN	127.1	113.3	17	NR
50	61	Abi, Enza, Taxane	Low	Para-aortic LN	60.2	297.035	-23	NR
52	76	Abi	Low	Para-aortic LN	249.8	99.65	-20	R
53	75	Other AR-targeted	Low	Supraclavicular LN	40.0	14.7	10	R

*Per criteria used in the CHAARTED clinical trial of the presence of visceral metastases or ≥4 bone lesions with ≥1 outside the vertebral bodies and pelvis.

ID, study patient identification number. Abi, abiraterone. Enza, enzalutamide. AR, androgen receptor. LN, lymph node. PSA, prostate-specific antigen, NR, non-responder. R, responder.

Supplemental Figures

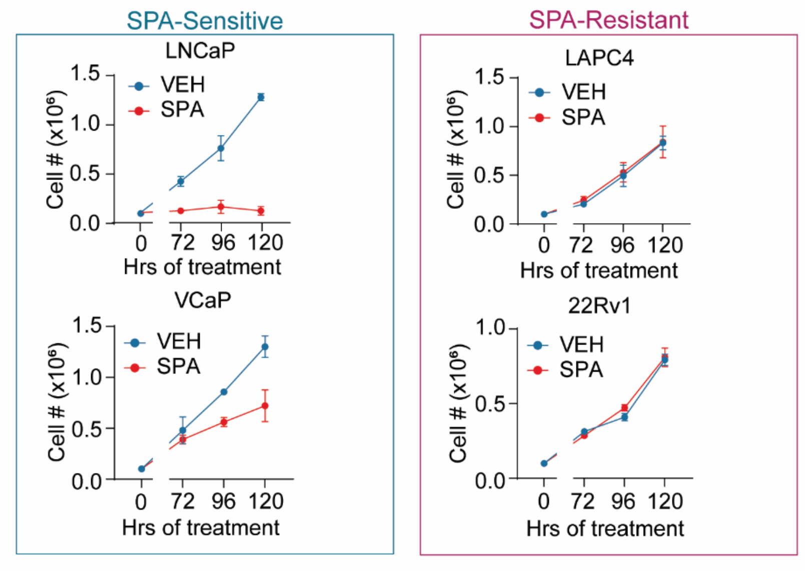

Figure S1. LNCaP and VCaP are SPA-sensitive prostate cancer cell lines, while LAPC4 and 22Rv1 are SPA-resistant cell lines. LNCaP, VCaP, LAPC4, and 22Rv1 viable cell number following treatment with VEH or SPA. p value by unpaired two-tailed t-test comparing final cell counts (n = 2 independent experiments).

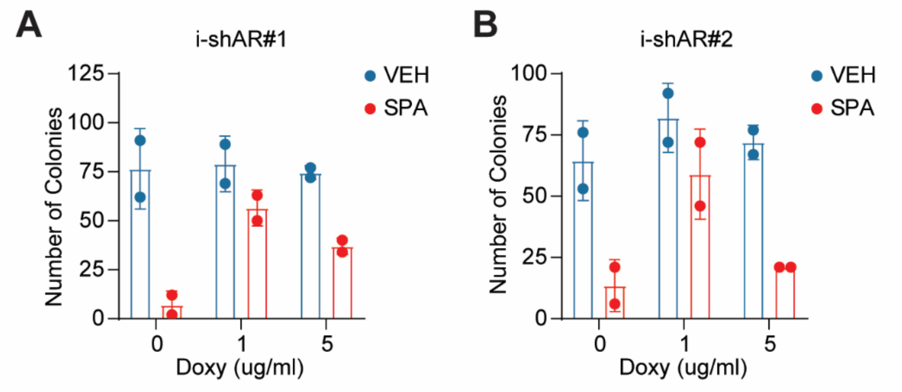

Figure S2. AR knock-down rescues clonogenic survival of LNCaP treated with SPA. Clonogenic survival of LNCaP-shAR pretreated with indicated concentration of doxycycline for 72 hours then VEH or SPA for 96 hours (n=2 independent experiments). VEH, vehicle control, EtOH 0.01%. SPA, R1881 10nM.

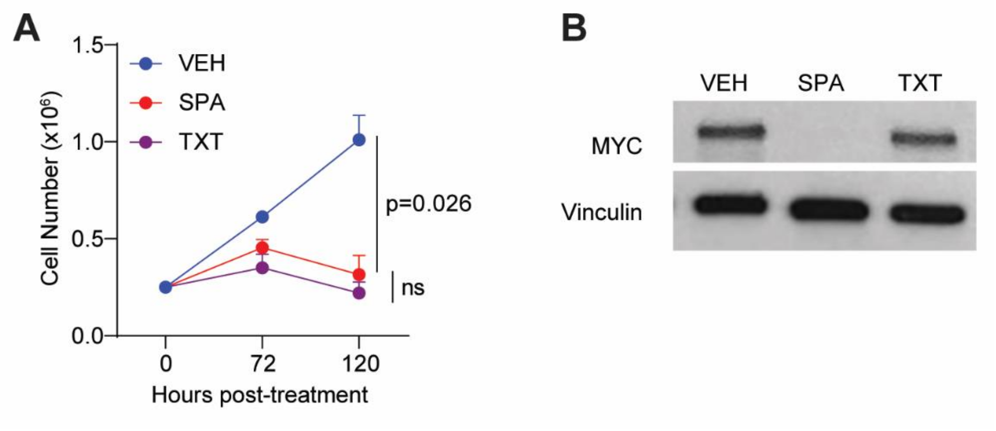

Figure S3. Downregulation of MYC is specific to growth inhibition by SPA. (**A**) LNCaP viable cell number following treatment with VEH, SPA, or TXT. p value by unpaired two-tailed t-test comparing final cell count (n=3 independent experiments). (**B**) MYC protein expression by western blof of LNCaP cells treated with VEH, SPA, or TXT for 72 hours. Representative blot of n=2 independent experiments. VEH, vehicle control, EtOH 0.01%. SPA, R1881 10nM. TXT, docetaxel 10uM.

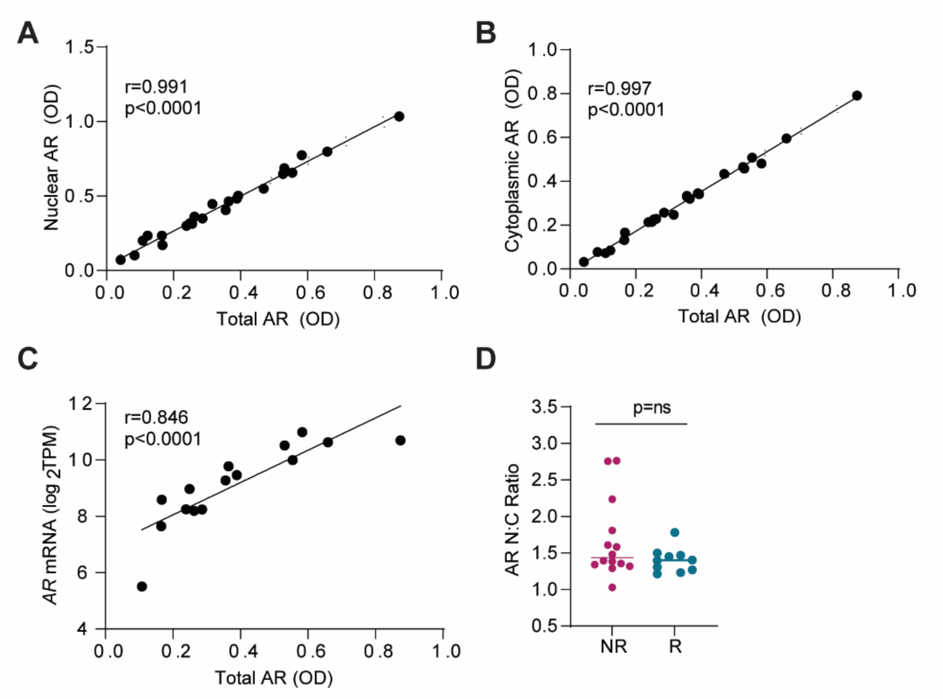

Figure S4. Total AR protein abundance correlates with nuclear AR, cytoplasmic AR, and AR mRNA in patient biopsies. (**A**) Correlation of nuclear AR with total AR within pre-BAT biopsies (n=24). (**B**) Correlation of cytoplasmic AR with total AR within pre-BAT biopsies (n=24). (**C**) Correlation of *AR* mRNA with total AR within pre-BAT biopsies (n=15). (**D**) Pre-BAT AR nuclear-to-cytoplasmic ratio (N:C) stratified by the presence or absence of a clinical response on C4D1 for samples adequate for IHC (n=24). p value by unpaired two-tailed t-test. (**A**-**C**) r and p values by Pearson's correlation calculation.

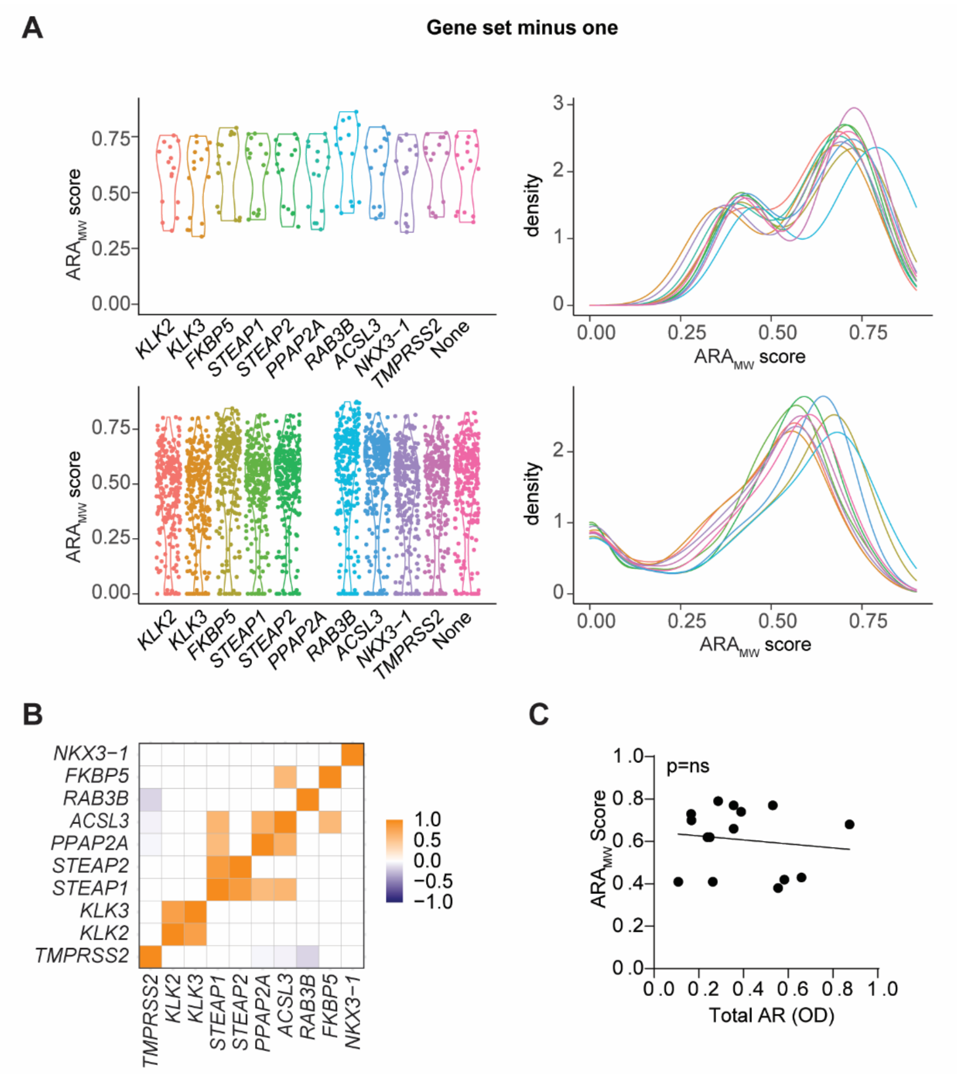

Figure S5. ARA$_{MW}$ score integrates expression of a panel of AR target genes and does not correlate with AR protein expression. (**A**) Distribution of ARA$_{MW}$ scores in the COMBAT pre-BAT mCRPC (n=15) and SU2C/PCF mCRPC (n=266) cohorts subtracting one gene from the gene set for score calculation. (**B**) Similarity matrix of gene expression of genes used in the ARA$_{MW}$ score among patients in the COMBAT pre-BAT mCRPC cohort (n=15). Scale is Pearson correlation coefficient. (**C**) Correlation of ARA$_{MW}$ score with AR protein expression among patients in the COMBAT pre-BAT mCRPC cohort (n=15). p value by Pearson's correlation calculation.

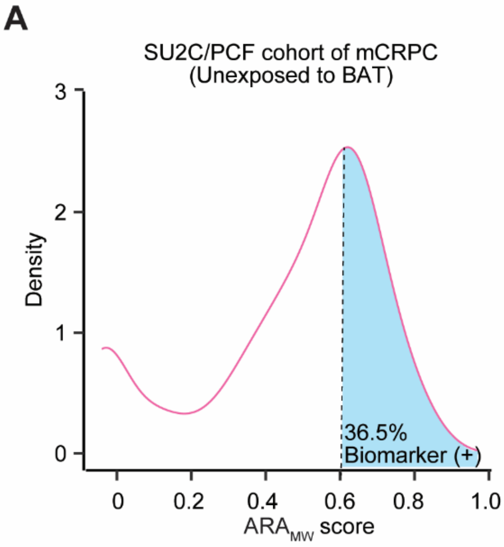

B

Altered Gene	Biomarker (-) (n=169)	Biomarker (+) (n=97)	p value
TMPRSS2-ERG	67 (39.6)	38 (39.2)	0.949
TP53	64 (37.9)	36 (37.1)	0.897
AR-amp	35 (20.7)	17 (17.5)	0.527
AR-mt	25 (14.8)	23 (23.7)	0.070
BRCA2	17 (10.1)	7 (7.2)	0.628
PTEN	14 (8.3)	5 (5.2)	0.346
APC	11 (6.5)	9 (9.3)	0.406
CTNNB1	11 (6.5)	2 (2.1)	0.110
CDK12	8 (4.7)	9 (9.3)	0.140
SPOP	8 (4.7)	5 (5.2)	0.856
ATM	7 (4.1)	8 (8.2)	0.162
BRCA1	7 (3.6)	3 (3.1)	0.829
RB1	6 (3.6)	6 (6.2)	0.328
BARD1	4 (2.4)	1 (1)	0.420
ARID1A	2 (1.2)	6. (6.2)	0.022

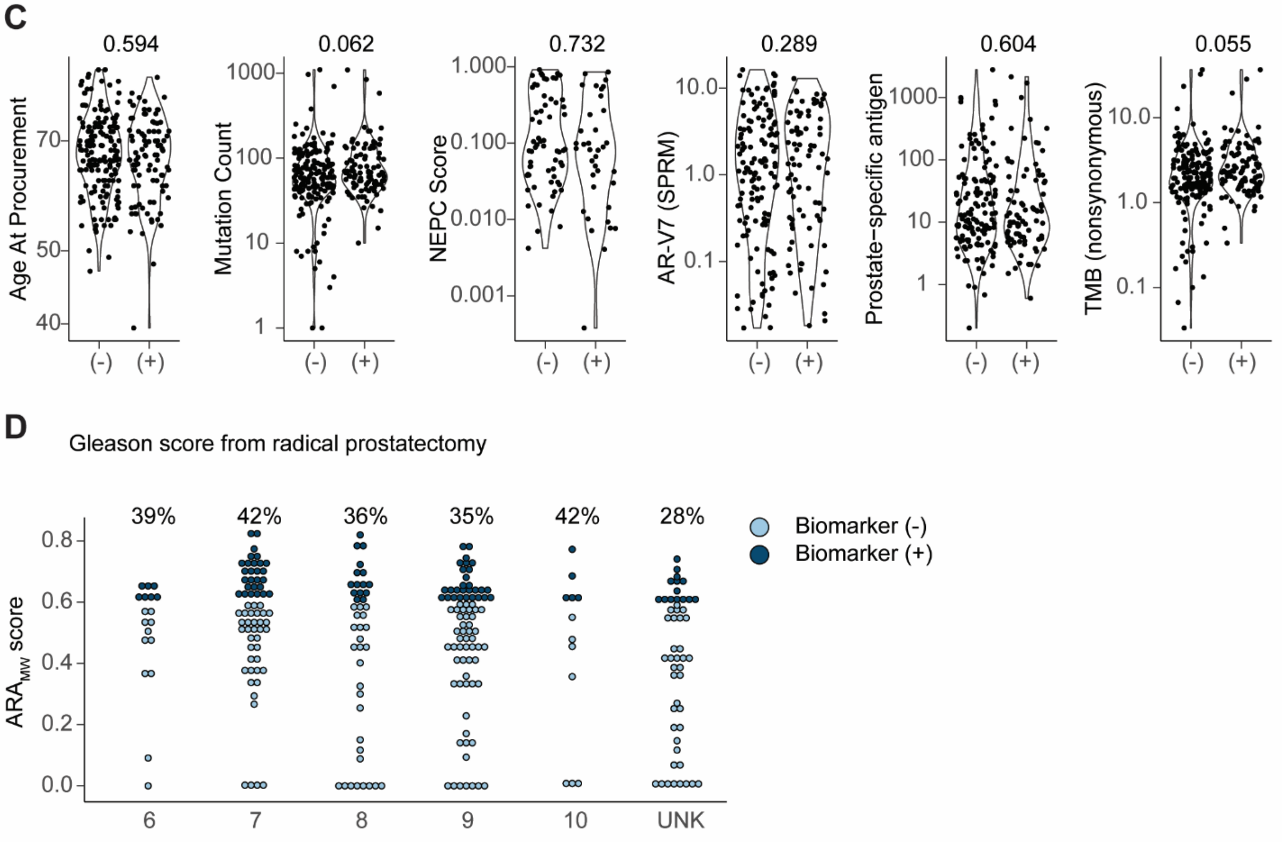

Figure S6. Characterization of the ARA$_{MW}$ score. (**A**) Distribution of ARA$_{MW}$ score among patients in the SU2C/PCF cohort of mCRPC (n=266). Patients with a score greater than 0.6 assigned as biomarker-positive. (**B**) Tumor gene mutations in patient samples in the SU2C/PCF cohort stratified by presence or absence of the biomarker. p value by Chi-squared comparison of proportions. (**C**) Comparison of patient and tumor characteristics of patients in the SU2C/PCF cohort stratified by the presence or absence of the biomarker. p value by Wilcoxon ranked sum test. (**D**) Gleason score of patients in the SU2C/PCF cohort stratified by the presence or absence of the biomarker. Biomarker (-), ARA$_{MW}$ score less than 0.6. Biomarker (+), ARA$_{MW}$ score greater than or equal to 0.6.

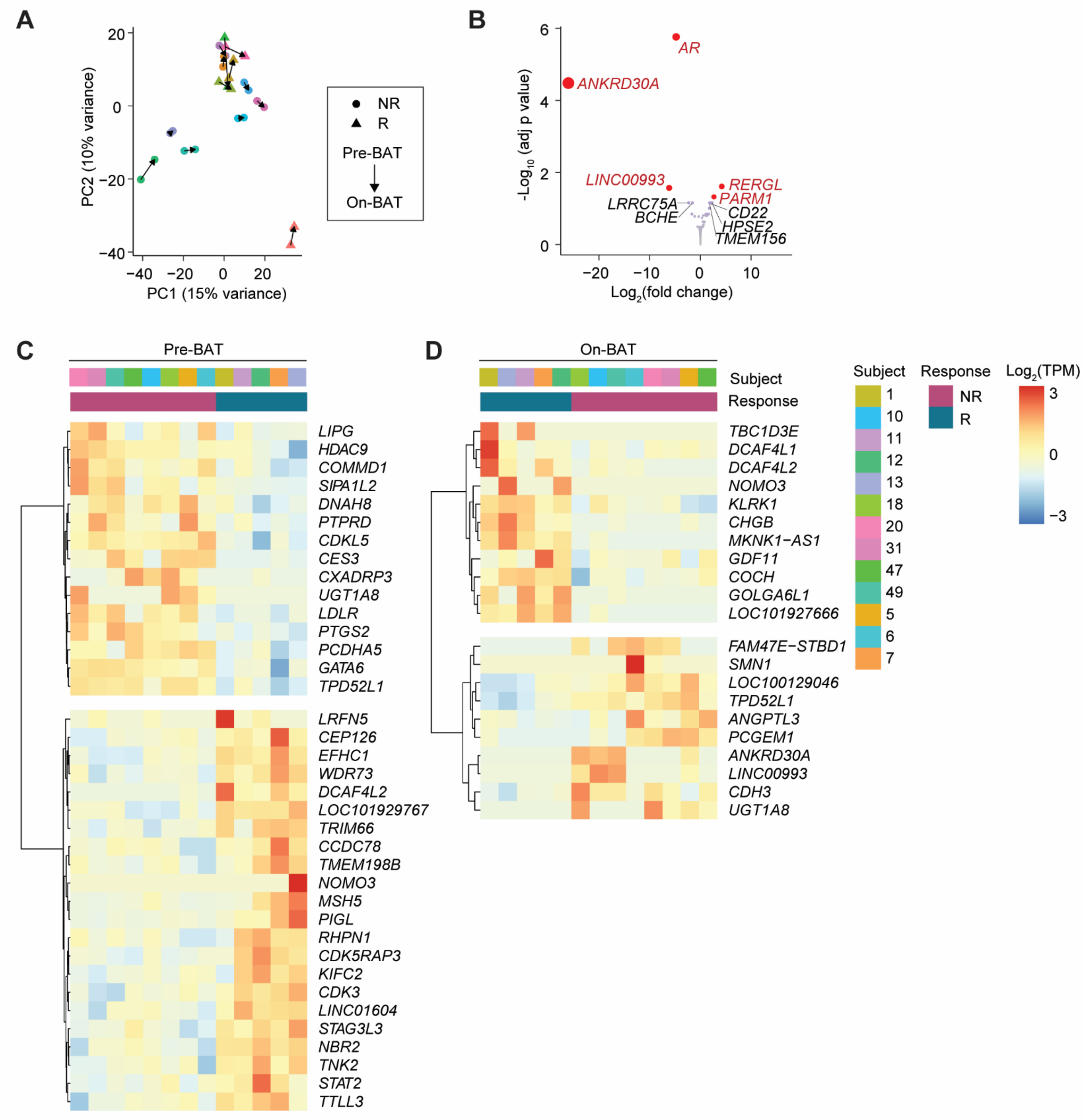

Figure S7. Unbiased analyses of RNA sequencing of paired patient biopsies. (**A**) Principal component (PC) analysis of RNA sequencing of patient biopsy samples. (**B**) Gene expression changes induced by BAT in paired patient biopsy samples. (**C-D**) Gene expression differences between non-responders (NR) and responders (R) prior to BAT (C) and on BAT (D). Only genes with an adjusted p value of less than 0.05 are shown.

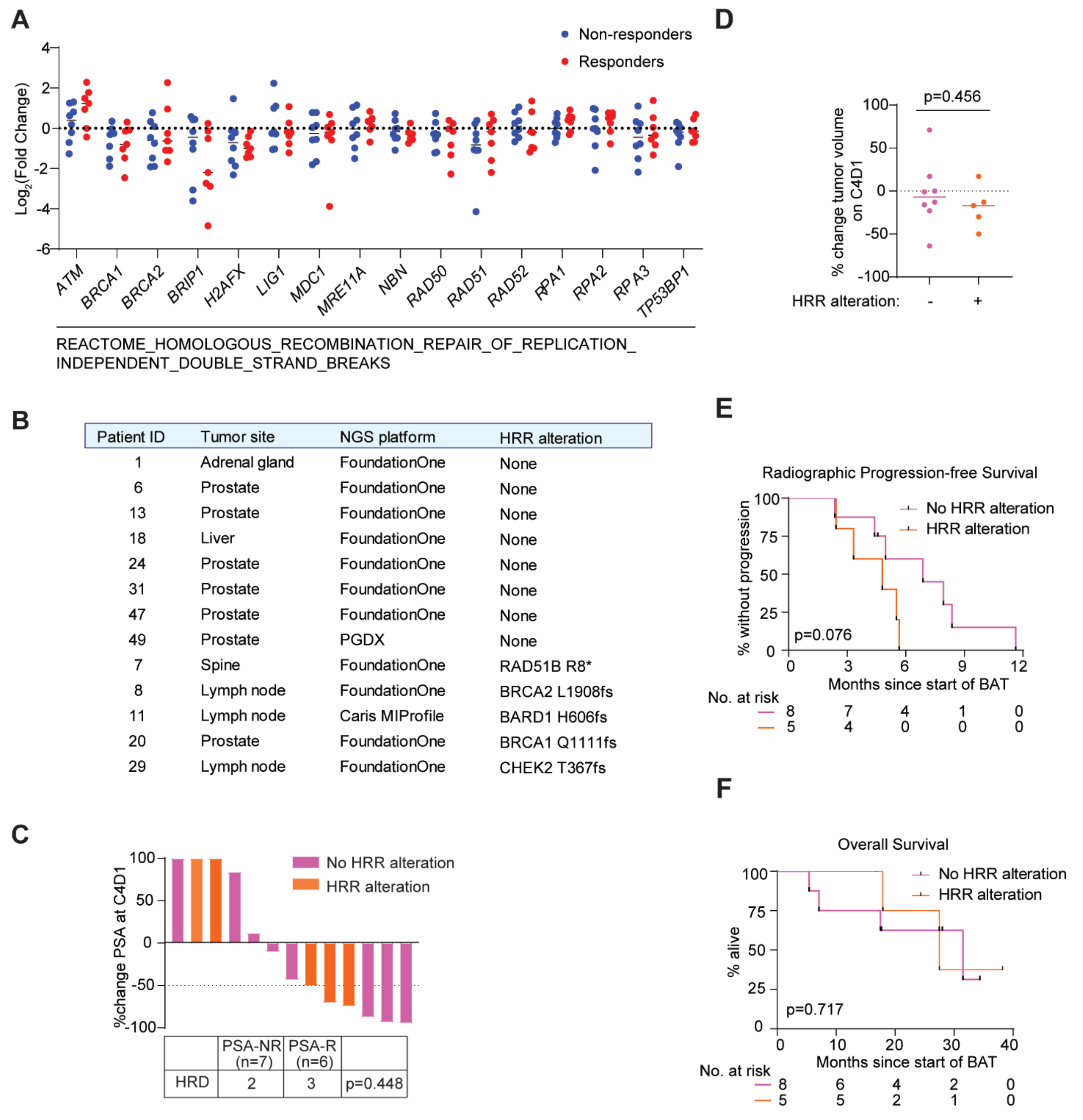

Patient ID	Tumor site	NGS platform	HRR alteration
1	Adrenal gland	FoundationOne	None
6	Prostate	FoundationOne	None
13	Prostate	FoundationOne	None
18	Liver	FoundationOne	None
24	Prostate	FoundationOne	None
31	Prostate	FoundationOne	None
47	Prostate	FoundationOne	None
49	Prostate	PGDX	None
7	Spine	FoundationOne	RAD51B R8*
8	Lymph node	FoundationOne	BRCA2 L1908fs
11	Lymph node	Caris MIProfile	BARD1 H606fs
20	Prostate	FoundationOne	BRCA1 Q1111fs
29	Lymph node	FoundationOne	CHEK2 T367fs

Figure S8. Homologous recombination repair (HRR) gene alterations do not predict response. (**A**) Change in HRR gene expression on BAT stratified by response (n=7 responders and 8 non-responders). (**B**) Clinical somatic next-generation sequencing (NGS) was performed for 13 patients prior to enrollment. (**C**) Percent change in PSA on C4D1 color-coded by the presence or absence of a somatic HRR alteration. PSA_{50} response indicated by dashed line. p value by Chi-squared comparison of proportions. (**D**) Percent change in tumor volume on C4D1 stratified by the presence or absence of an HRR alteration. p value by unpaired t test. (**E**) Radiographic progression-free survival on BAT stratified by the presence or absence of an HRR alteration. p value by log-rank. (**F**) Overall survival on BAT stratified by the presence or absence of an HRR alteration. p value by log-rank.

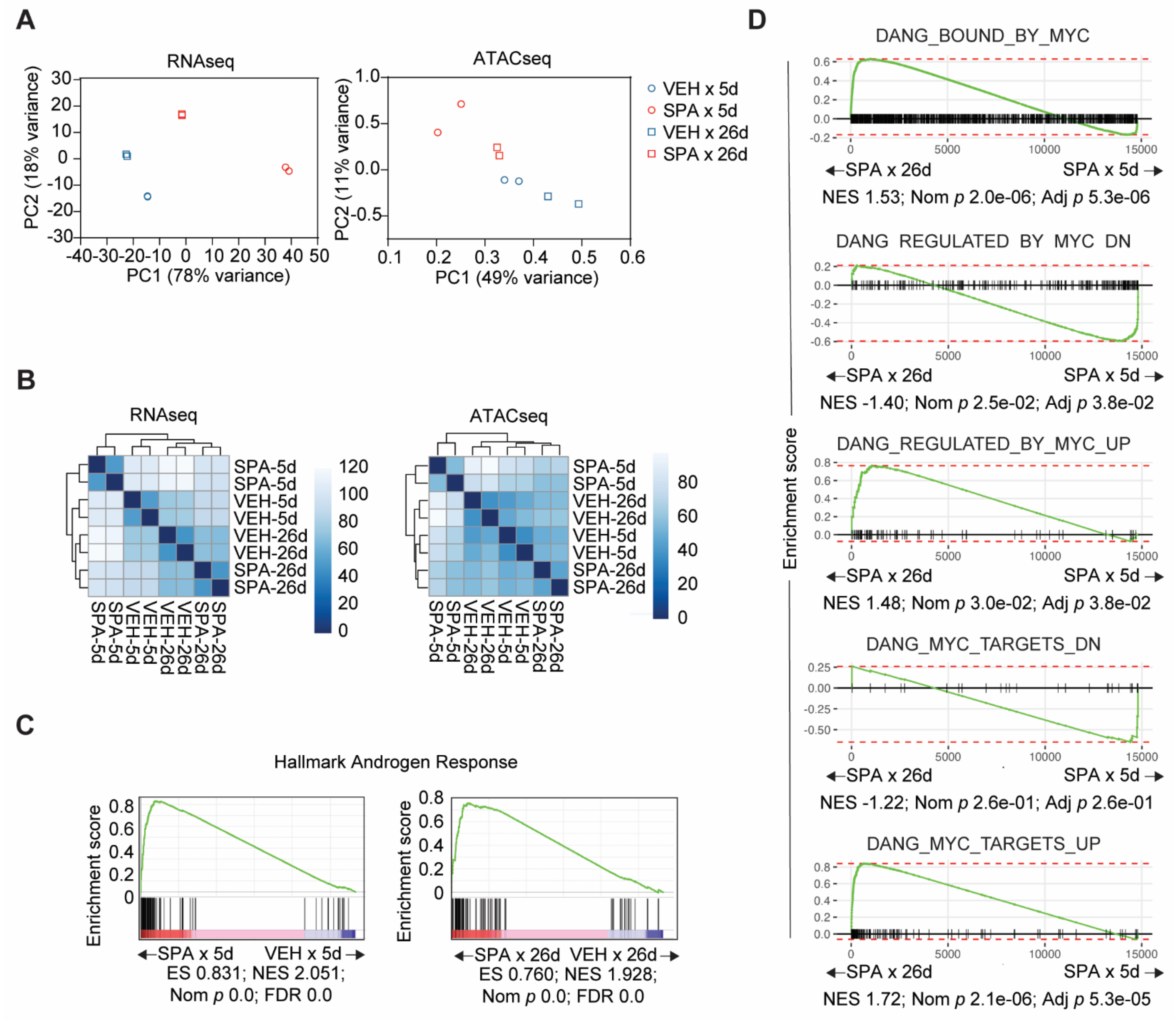

Figure S9. Cells with acquired resistance to SPA revert to a pre-treatment phenotype but maintain AR activation. Principal component (PC) analysis (**A**) and clustering analysis (**B**) of RNA sequencing and ATAC sequencing of LNCaP cells treated with VEH or SPA for 5 or 26 days. (**C**) Hallmark Androgen Response gene set enrichment analysis tracings comparing LNCaP cells treated with VEH to SPA for 5 or 26 days. (**D**) Gene set enrichment analysis tracings using gene sets previously determined to be regulated by MYC comparing LNCaP cells treated with SPA for 5 or 26 days. ES, enrichment score; NES normalized enrichment score; FDR, false discovery ratio.

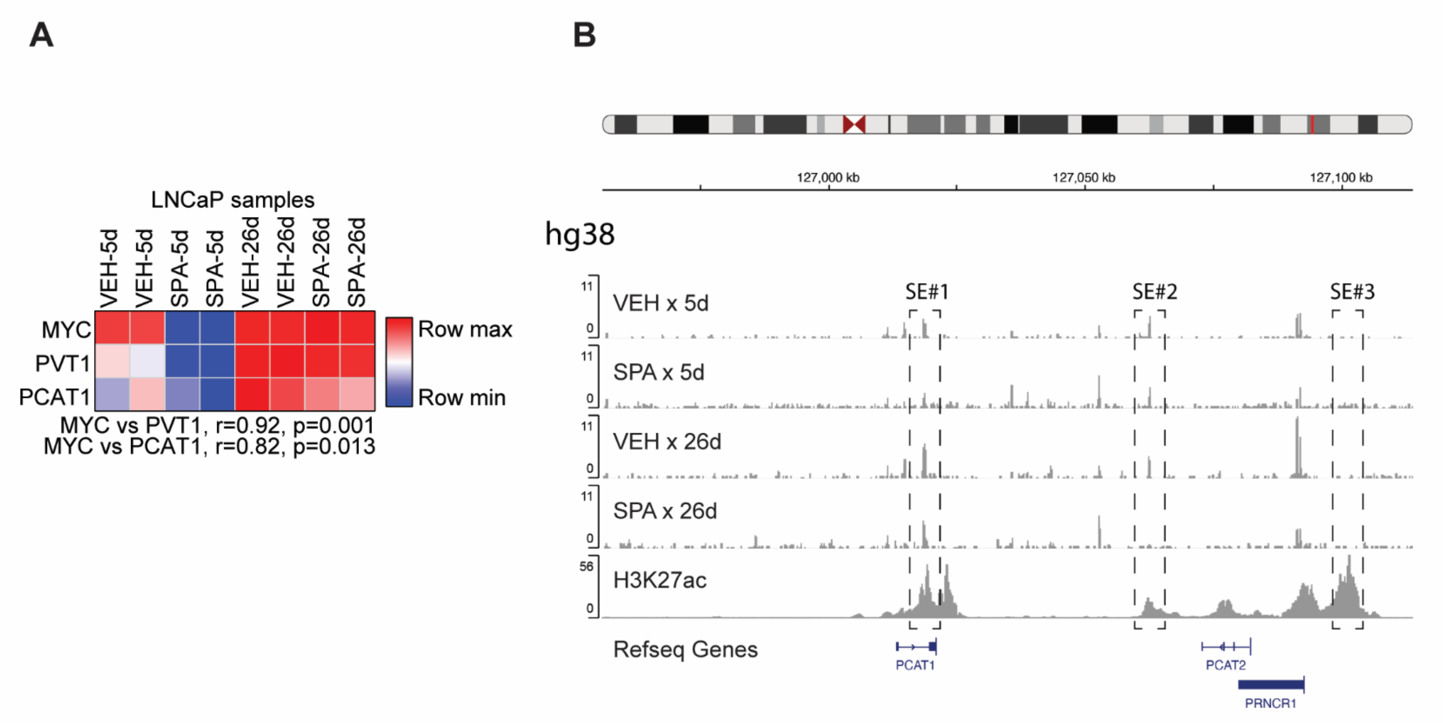

Figure S10. Acquired resistance to SPA is associated with an alteration in superenhancer activity on 8q24. (**A**) Expression of genes within the 8q24 topologically associated domain (TAD) in LNCaP cells treated with VEH or SPA for 5 and 26 days. r and p values by Pearson's correlation calculation. (**B**) Chromatin accessibility of the MYC super-enhancers (SE) of LNCaP cells treated with VEH or SPA for 5 or 26 days. H3K27ac CHIP-seq on VCaP obtained from the ENCODE project. VEH, vehicle control, EtOH 0.01%. SPA, R1881 10nM.

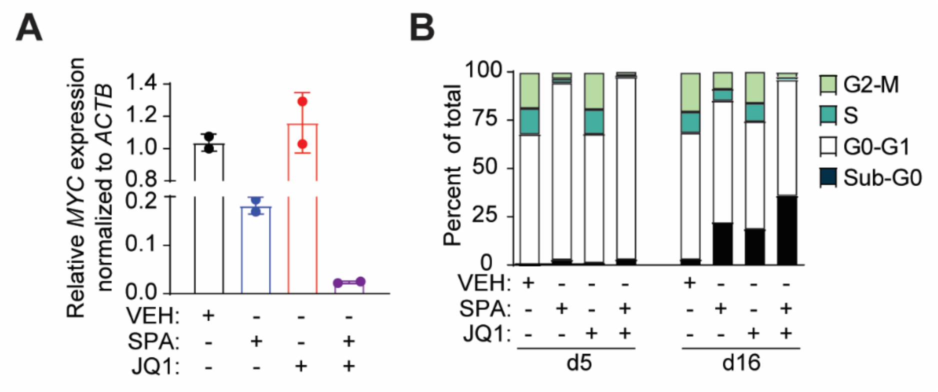

Figure S11. Dual inhibition of MYC by SPA and JQ1 prolongs growth inhibition of LNCaP cells. (**A**) MYC mRNA expression by qPCR of LNCaP cells treated with VEH, SPA, JQ1 50nM, or SPA and JQ1 for 72 hours (n=2 independent experiments). (**B**) Cell cycle analysis by propidium iodide staining of LNCaP treated with VEH, SPA, JQ1 50nM, or SPA and JQ1. Average values of n=2 experiments. VEH, vehicle control, EtOH 0.01%. SPA, R1881 10nM.

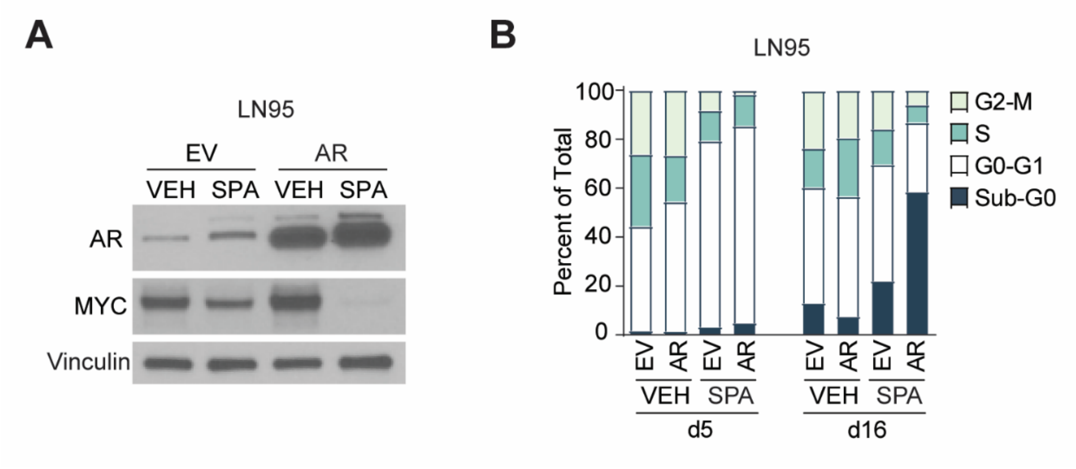

Figure S12. Constitutive expression of AR in LN95 cells reduces acquired resistance to SPA. (**A**) AR and MYC protein expression by western blot of LN95-EV and LN95-AR cells treated with VEH or SPA for 72 hours. Representative blot of n=3 independent experiments. Vinculin used as a loading control. (**B**) Cell cycle analysis by propidium iodide staining of LN95-EV and LN95-AR cells treated with VEH or SPA. Average values of n=3 independent experiments. VEH, vehicle control, EtOH 0.01%. SPA, R1881 10nM.

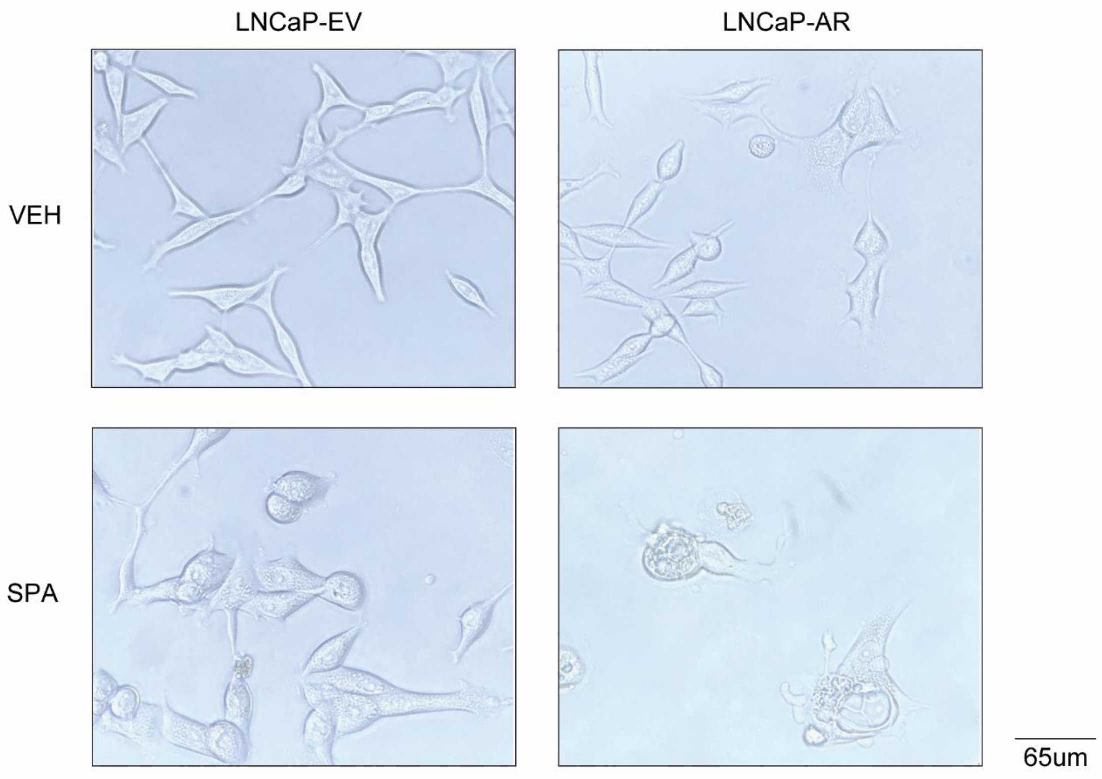

Figure S13. SPA results in extensive vacuolization in LNCaP cells with constitutively high AR expression. Representative photographs of light microscopy of LNCaP-EV and LNCaP-AR treated with VEH or SPA for 5 days. VEH, vehicle control, EtOH 0.01%. SPA, R1881 10nM.

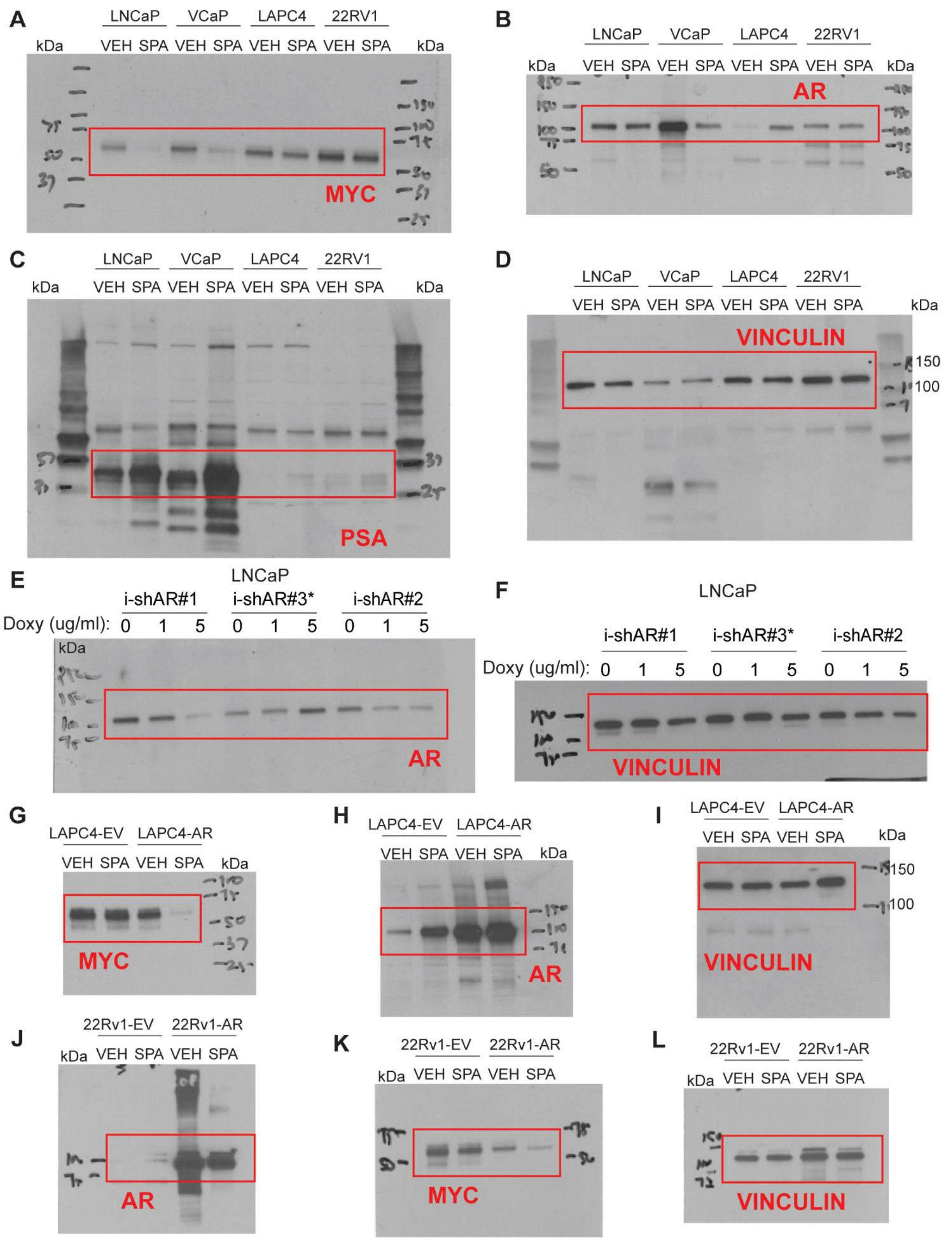

Figure S14. Full western blots for Figure 1. The full, uncut blot images of Figure 1 are shown (A-D correspond to Fig 1A; E-F correspond to Fig 1B; G-I correspond to Fig 1D; J-L correspond to Fig 1F). Vinculin is used as a loading control (D, F, I, L). LNCaP expressing i-shAR#3 (*) in E did not result in AR knock-down and these cells are not described in the manuscript.

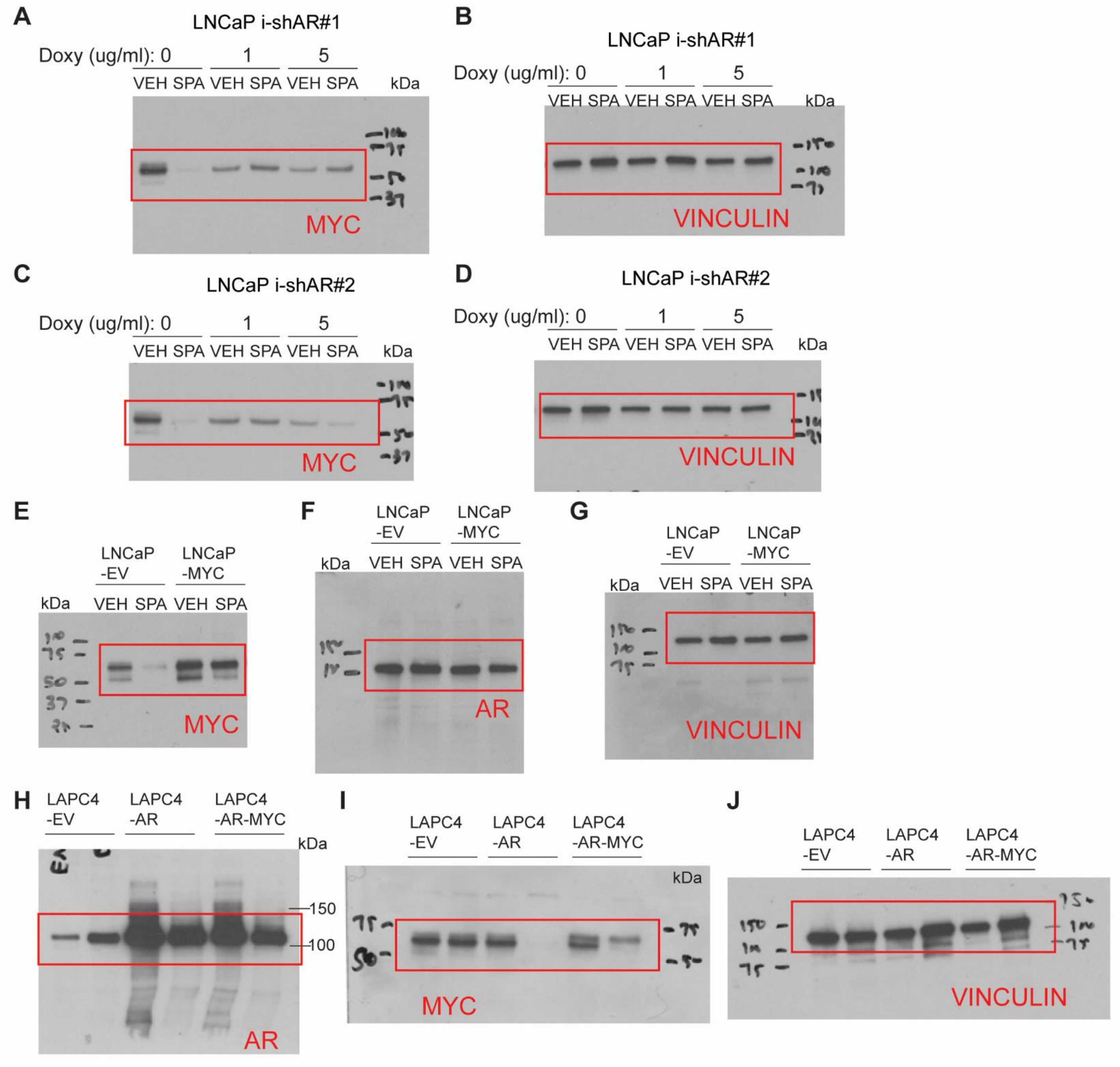

Figure S15. Full western blots for Figure 2. The full, uncut blot images of Figure 2 are shown (A-D correspond to Fig 2B; E-G correspond to Fig 2C; H-J correspond to Fig 2E). Vinculin is used as a loading control (B, D, G, J).

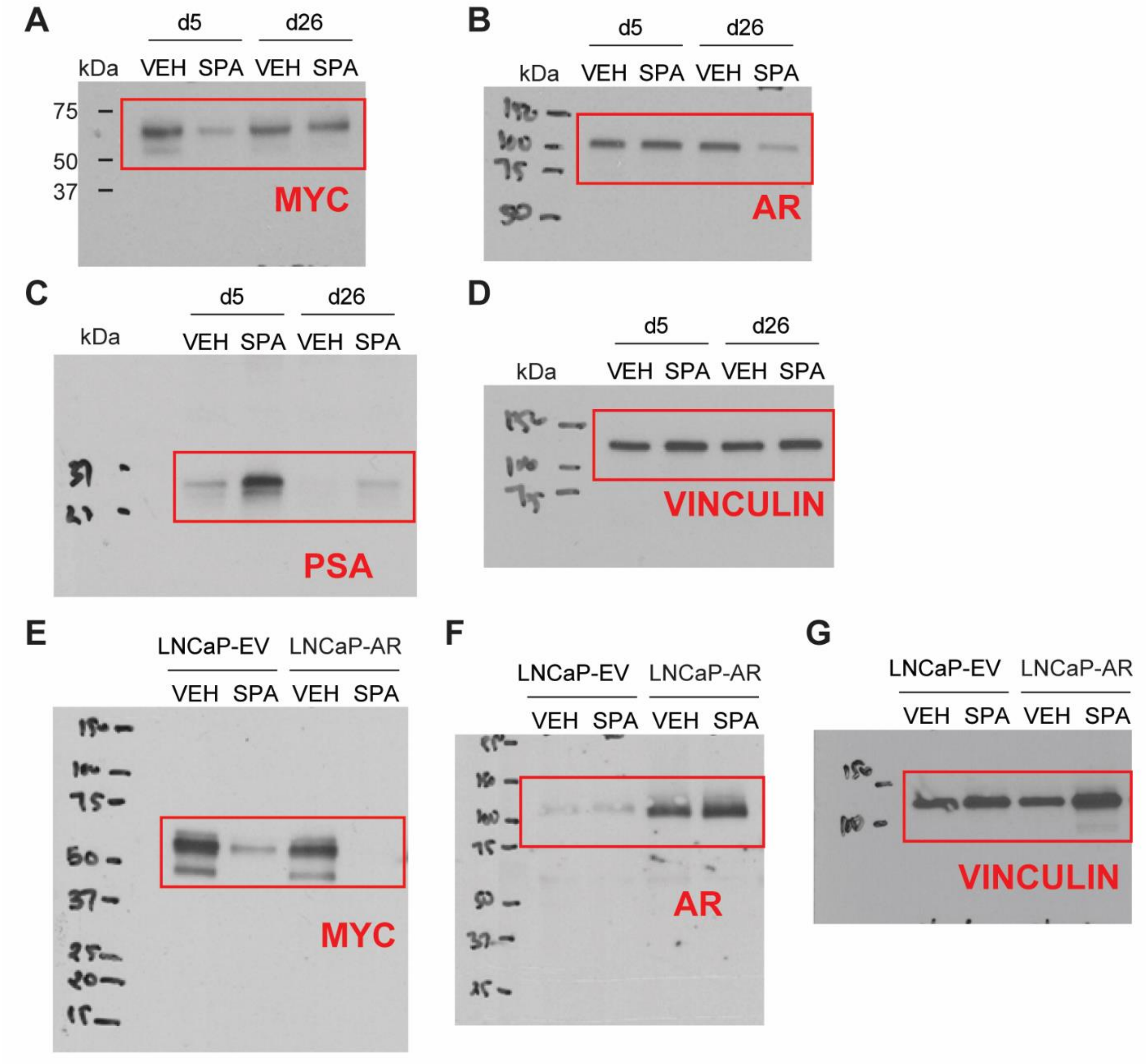

Figure S16. Full western blots for Figure 5. The full, uncut blot images of Figure 5 are shown (A-D correspond to Fig 5F; E-G correspond to Fig 5H). Vinculin is used as a loading control (D, G).

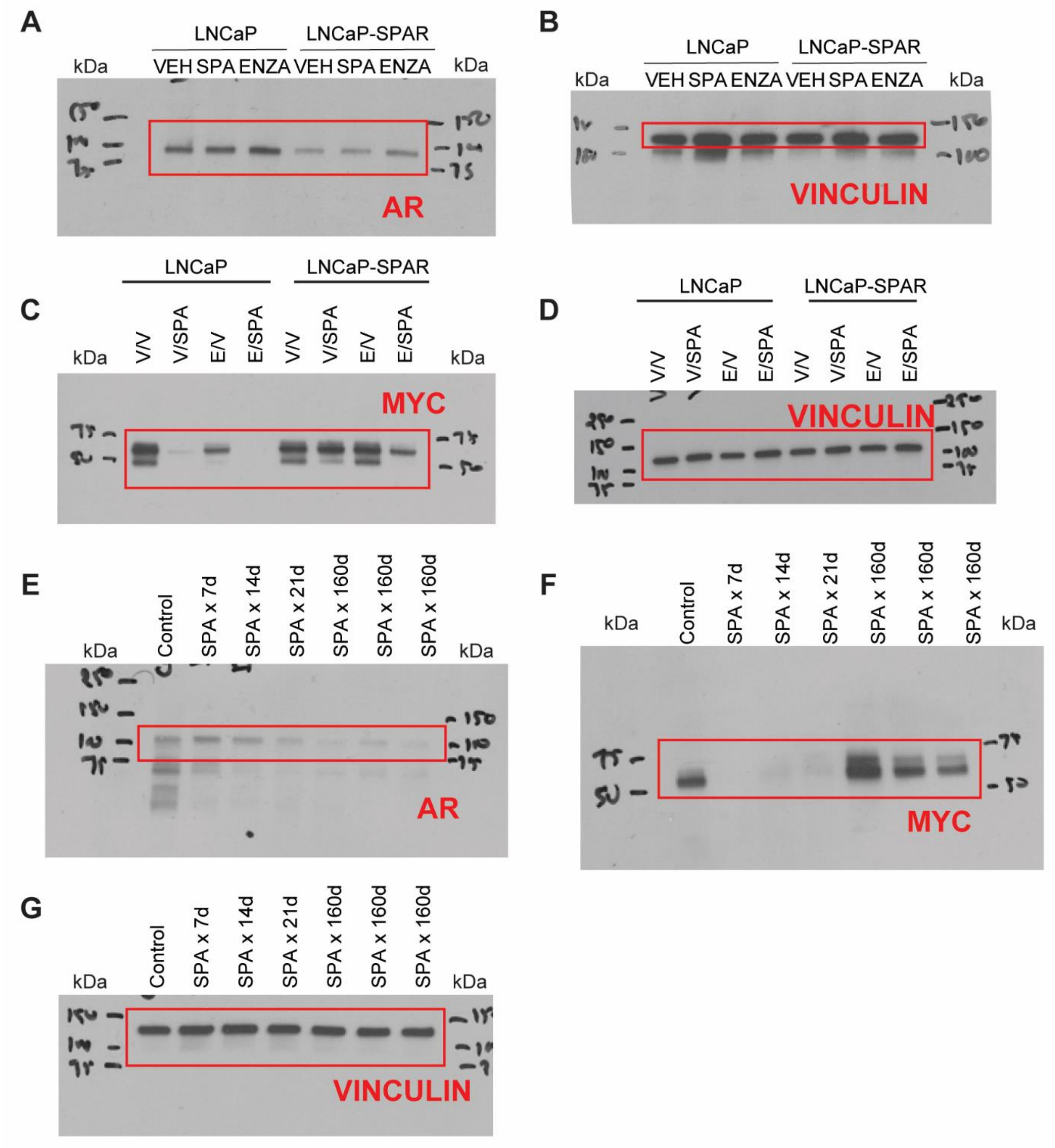

Figure S17. Full western blots for Figure 7. The full, uncut blot images of Figure 7 are shown (A-B correspond to Fig 7C; C-D correspond to Fig 7D, E-G correspond to Fig 7G). Vinculin is used as a loading control (B, D, G).

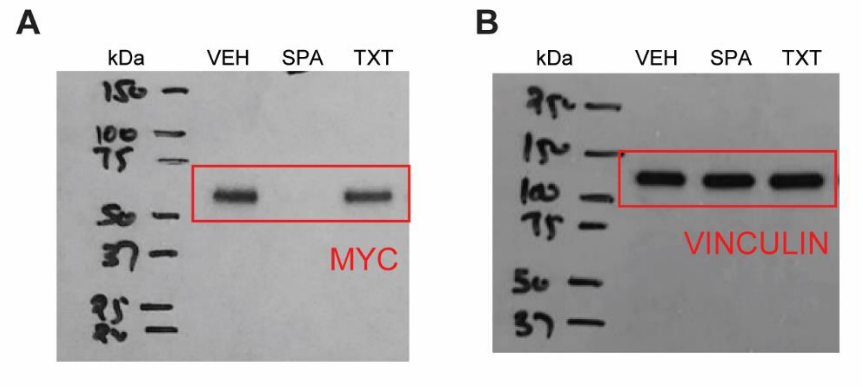

Figure S18. Full blots for Figure S3B. The full, uncut blot images of Supplementary Figure 3B are shown. Vinculin is used as a loading control (B).

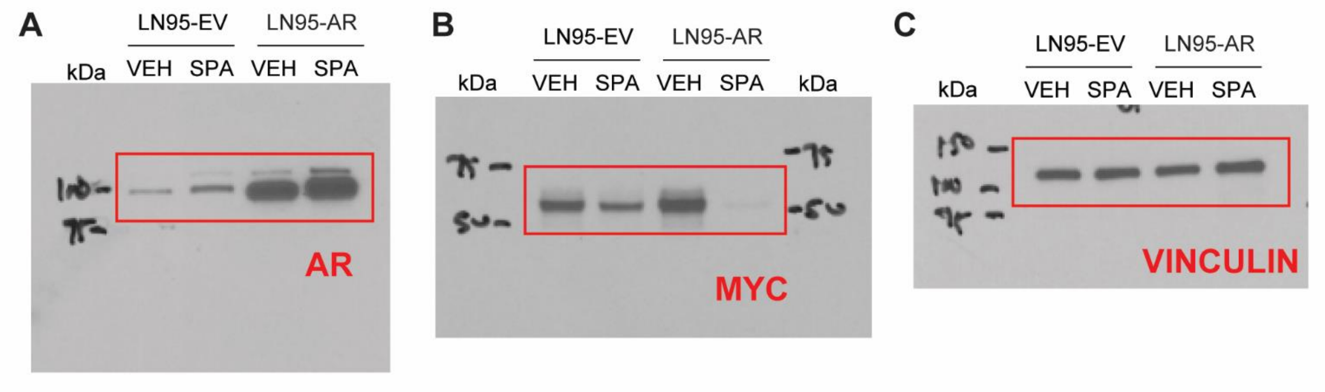

Figure S19. Full western blots for Figure S12A. The full, uncut blot images of Supplementary Figure 12A are shown. Vinculin is used as a loading control (C).
